# Molecular recognition of surface-immobilized carbohydrates by a synthetic lectin

**DOI:** 10.3762/bjoc.10.138

**Published:** 2014-06-16

**Authors:** Melanie Rauschenberg, Eva-Corrina Fritz, Christian Schulz, Tobias Kaufmann, Bart Jan Ravoo

**Affiliations:** 1 Organic Chemistry Institute, Westfälische Wilhelms-Universität Münster, Corrensstrasse 40, 48149 Münster, Germany

**Keywords:** carbohydrates, lectins, molecular recognition, microarrays, multivalent glycosystems, peptides

## Abstract

The molecular recognition of carbohydrates and proteins mediates a wide range of physiological processes and the development of synthetic carbohydrate receptors (“synthetic lectins”) constitutes a key advance in biomedical technology. In this article we report a synthetic lectin that selectively binds to carbohydrates immobilized in a molecular monolayer. Inspired by our previous work, we prepared a fluorescently labeled synthetic lectin consisting of a cyclic dimer of the tripeptide Cys-His-Cys, which forms spontaneously by air oxidation of the monomer. Amine-tethered derivatives of *N-*acetylneuraminic acid (NANA), β-D-galactose, β-D-glucose and α-D-mannose were microcontact printed on epoxide-terminated self-assembled monolayers. Successive prints resulted in simple microarrays of two carbohydrates. The selectivity of the synthetic lectin was investigated by incubation on the immobilized carbohydrates. Selective binding of the synthetic lectin to immobilized NANA and β-D-galactose was observed by fluorescence microscopy. The selectivity and affinity of the synthetic lectin was screened in competition experiments. In addition, the carbohydrate binding of the synthetic lectin was compared with the carbohydrate binding of the lectins concanavalin A and peanut agglutinin. It was found that the printed carbohydrates retain their characteristic selectivity towards the synthetic and natural lectins and that the recognition of synthetic and natural lectins is strictly orthogonal.

## Introduction

In comparison to proteins and nucleic acids, carbohydrates have traditionally received less attention in the scientific community. However, it is increasingly apparent that carbohydrates and glycoconjugates are involved in a multitude of physiological and pathological processes and offer an enormous potential to encrypt biological information [1–4]. In contrast to the linear nucleic acids and proteins, carbohydrates usually form branched oligomers or polymers which are joined together by a variety of linkages. The vast amount of resulting carbohydrate oligomers and polymers offers a virtually unlimited number of encodings. In nature, carbohydrates are often linked to lipids, peptides or proteins. These conjugates are found inside cells, on cell membranes as well as in extracellular fluids and matrices. Surprisingly, the general understanding of the function of carbohydrates in cell biology is still lagging far behind our knowledge of proteins and nucleic acids. This backlog is mainly due to the complexity of biological carbohydrates. Additionally, established analytical and synthetic methods in protein and nucleic acid research such as automated sequencing, automated synthesis and high-throughput microarray screening are lacking in glycobiology [5]. However, during the last decade, these methods have also been adapted to carbohydrates [6–8].

Carbohydrate microarrays on chips proved to be a particularly useful tool in glycomics [9–11] since their description in 2002 by several groups [12–17]. Microarrays normally consist of carbohydrates immobilized in an ordered and well defined format on a flat surface. The arrays can be considered as minimal models of cell surfaces that are compatible with high-throughput analysis techniques and can be used to study carbohydrate–protein/antibody interactions, to detect enzymes cleaving glycosidic bonds, to study cell–cell adhesions mediated by carbohydrates as well as to screen for selective inhibitors of carbohydrate–protein interactions [18]. The immobilization of carbohydrates can be divided into noncovalent and covalent attachment routes. While carbohydrates simply adhere to the surface when using the noncovalent strategy, the covalent attachment leads to the fabrication of highly stable arrays because a chemical bond is formed between the substrate and the carbohydrates. Fabrication of carbohydrate microarrays can be achieved either by microspotting of carbohydrates on activated surfaces or by using printing techniques on activated substrates. The first approach can be realized by robotic printers [19–20] generating high density chips with a large number of different spots. Read out is performed with an array scanner using fluorescence microscopy or surface plasmon resonance.

In recent years, microcontact printing (μCP) [21–24] has gained importance as a replication method for biological microarrays such as protein [25–26] and DNA microarrays [27–30]. Our group has shown that also simple carbohydrate microarrays can be conveniently prepared by μCP if reactive glycosides are printed on a suitable target self-assembled monolayer (SAM) [31–32]. Amongst others, we reported carbohydrate microarrays fabricated by cycloaddition of alkynes on azide-terminated SAMs [33], by Diels–Alder reaction of cyclopentadienes and furans on maleimide-terminated SAMs [34], by thiol–ene click reaction of functionalized thiols on alkene-terminated SAMs [35] as well as by strain promoted cycloadditions on azide- and nitriloxide-terminated SAMs [36] using μCP. Homogenous spots, high-edge resolution, good reproducibility and short reaction times can be easily achieved by using µCP. These advantages render this method a versatile tool for the fabrication of simple carbohydrate microarrays.

**Figure 1:**
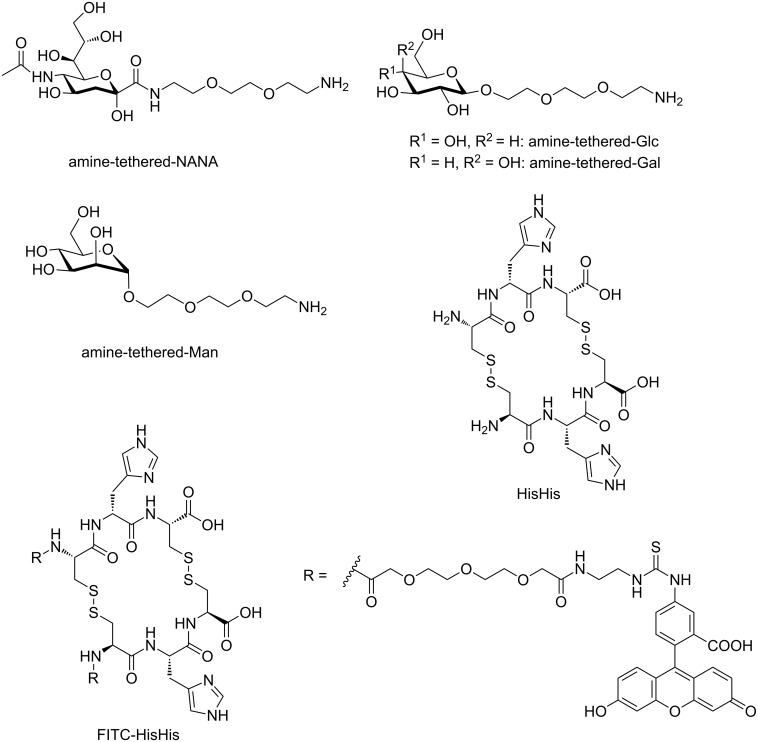
Molecular structures of carbohydrates (NANA, Glc, Gal, Man) immobilized on epoxide SAMs, NANA-binding synthetic lectin (HisHis), and the FITC-labeled synthetic lectin (FITC HisHis). For simplicity, only the parallel isomer of HisHis is shown.

“Synthetic lectins” as artificial carbohydrate receptors would be highly valuable as drugs in various therapies and as recognition units in diagnostics and sensing. However, the development of synthetic lectins poses a phenomenal challenge for supramolecular chemistry [37] because a carbohydrate receptor in order to be useful must not only compete with the strong hydration of carbohydrates in water but also discriminate closely related isomers. It is obvious that the de-novo design of a “synthetic lectin” is very difficult. The most remarkable synthetic lectins to date have been prepared by Davis and co-workers, who recently synthesized a cage-like receptor that binds glucose in water with excellent selectivity versus other simple carbohydrates (for example, ~50:1 versus galactose) which also has sufficient affinity for glucose sensing at the concentrations found in blood [38]. On the other hand, we have described a dynamic combinatorial approach to the identification of biomimetic carbohydrate receptors [39]. To this end, we explored a dynamic combinatorial library of cyclic peptides to select receptors that are assembled from tripeptides under thermodynamic equilibrium. Amongst others, we identified a synthetic lectin (HisHis) that binds *N*-acetylneuraminic acid (NANA) [18]. HisHis is a cyclic hexapeptide which is easily obtained from the air oxidation of the tripeptide Cys-His-Cys (see Figure 1). It should be noted that in this case HisHis is obtained as a mixture of two isomers, in which the tripeptides are either oriented in parallel or antiparallel direction. The parallel isomer is shown in Figure 1. We have recently synthesized and isolated the parallel and antiparallel isomers of HisHis and found that both isomers can bind NANA in a cooperative 1:2 complex (1 molecule of HisHis and 2 molecules of NANA) [40]. The binding constants are *K*_1_ = 143 M^−1^ and *K*_2_ = 5.08 × 10^3^ M^−1^ for the parallel isomer and *K*_1_ = 94 M^−1^ and *K*_2_ = 990 M^−1^ for the antiparallel isomer at neutral pH in water [40]. We also found that the parallel isomer of HisHis (but not the antiparallel isomer) binds β-D-galactose in a 1:1 complex with *K* = 7.96 × 10^3^ M^−1^ [40]. NMR spectroscopy and DFT calculations indicate that the interaction of the peptide with the carbohydrates is based primarily on hydrogen bonding [40]. It should be emphasized that NANA is a particularly interesting carbohydrate since it belongs to the class of sialic acids which are often the terminal carbohydrates in glycoproteins and glycolipids on the cell surface. Sialic acids are involved in the communication of cells with their environment [41] and selective detection, binding and blocking of sialic acids on the cell surface is of significant biomedical interest.

It is the aim of this report to investigate whether the synthetic lectin HisHis is able to selectively bind to NANA immobilized on a substrate which serves as a model for a cell surface. To this end, we prepared a set of simple carbohydrate microarrays as well as fluorescein-isothiocyanate labeled HisHis (FITC-HisHis) and studied the selectivity and affinity of HisHis towards immobilized NANA in comparison with the glycosides of glucose (Glc), galactose (Gal) and mannose (Man) (Figure 1). In this study, we exploit the epoxide ring opening reaction of amine-tethered carbohydrates on epoxide-terminated SAMs [42] to print carbohydrate microarrays on silicon and glass substrates. Epoxide-terminated SAMs are particularly versatile for the fabrication of biological arrays [43–44] and we have recently demonstrated that epoxide-terminated substrates are easily modified using µCP [45]. The incubation of the synthetic lectin FITC-HisHis as well as two natural lectins on the immobilized carbohydrates provides insight into the affinity and selectivity of lectin–carbohydrate interaction.

## Results and Discussion

The synthetic lectin HisHis was prepared by air oxidation of the tripeptide Cys-Hys-Cys (synthesized by solid phase peptide synthesis) as described previously [39]. Fluorescein-labeled FITC-HisHis was obtained by labeling of Cys-His-Cys with fluorescein isothiocyanate, which was achieved by using an Fmoc-protected oligo(ethylene glycol) spacer synthesized in four steps from commercially available ethylenediamine (see Supporting Information File 1). The introduction of this water soluble spacer should ensure the unhindered formation of the cyclic synthetic lectin FITC-HisHis from the FITC-labeled Cys-His-Cys by air oxidation. The spectroscopic and analytical data obtained for FITC-HisHis are fully consistent with the molecular structure (see Supporting Information File 1). Successful incorporation of the fluorophore was also evident in the UV–vis spectrum of the precursor FITC-labeled Cys-His-Cys (see Figure S1 in the Supporting Information File 1). We note that if prepared directly by air oxidation from Cys-His-Cys, HisHis as well as FITC-HisHis consist of a mixture of two isomers, in which the two tripeptides are arranged in parallel or antiparallel direction, respectively. We have recently synthesized and isolated the parallel and antiparallel HisHis isomers and found that both isomers can bind NANA in a 1:2 complex with slightly stronger binding by the parallel HisHis compared to the antiparallel HisHis [40]. Isothermal titration calorimetry (ITC) confirmed that the interaction of FITC-HisHis (mixture of isomers) and NANA is characterized by the same stoichiometry (1:2) and nearly the same binding constants (*K*_1_ = 163 M^−1^ and *K*_2_ = 5.36 × 10^3^ M^−1^) were obtained. ITC data are provided in Supporting Information File 1. These findings indicate that the introduction of the FITC label does not affect the formation of the synthetic lectin HisHis and its interaction with NANA. Since the mixture of isomers is much more easily synthesized than the individual isomers and since both isomers are potent binders of NANA, all experiments described in this report where performed with the mixture of parallel and antiparallel isomers of FITC-HisHis.

In order to study the affinity of the synthetic lectin HisHis on the surface, four carbohydrates (NANA, Glc, Gal, Man, see Figure 1) were selected for the fabrication of carbohydrate arrays. To provide carbohydrate inks suitable for microcontact printing (µCP), NANA was conjugated via its C1 carboxylic acid moiety, whereas the other carbohydrates were conjugates as β-glycosides (Glc, Gal) and α-glycosides (Man), respectively. In order to flexibly attach the carbohydrates on the substrate and to ensure unhindered carbohydrate–lectin interactions, oligo(ethylene glycol) spacers were introduced. Oligo(ethylene glycol) chains are flexible, water-soluble and do not interact with lectins [46]. A nucleophilic primary amine function was introduced on the terminus of the spacer in order to ensure reaction with the epoxide-functionalized substrate upon µCP. The NANA ink was prepared by solution phase peptide coupling using the DIPCDI/Oxyma pure^®^ coupling protocol. The glucose (Glc), galactose (Gal) and mannose (Man) inks were synthesized as described in Supporting Information File 1.

The effective immobilization of the amine-terminated carbohydrate inks requires a surface functionalized with epoxides. Epoxides are stable under ambient conditions yet highly reactive towards amines at elevated temperatures and epoxide SAMs on silicon oxide surfaces can be obtained in only one step from an epoxy-terminated trimethoxysilane. The most common approaches to fabricate epoxide SAMs is by dip-coating or vapor condensation. However, this leads to the formation of thick films of aggregated molecular layers with undefined surface morphology [47–50]. Therefore, the epoxide SAMs were prepared by the method of Julthongpiput et al. because well-defined monolayers of epoxides on glass and silicon substrates can be obtained by using (3-glycidoxypropyl)trimethoxysilane in toluene [42]. The successful surface modification was verified by contact angle and XPS measurements (see Supporting Information File 1) which are in accordance with literature data [42].

**Figure 2:**
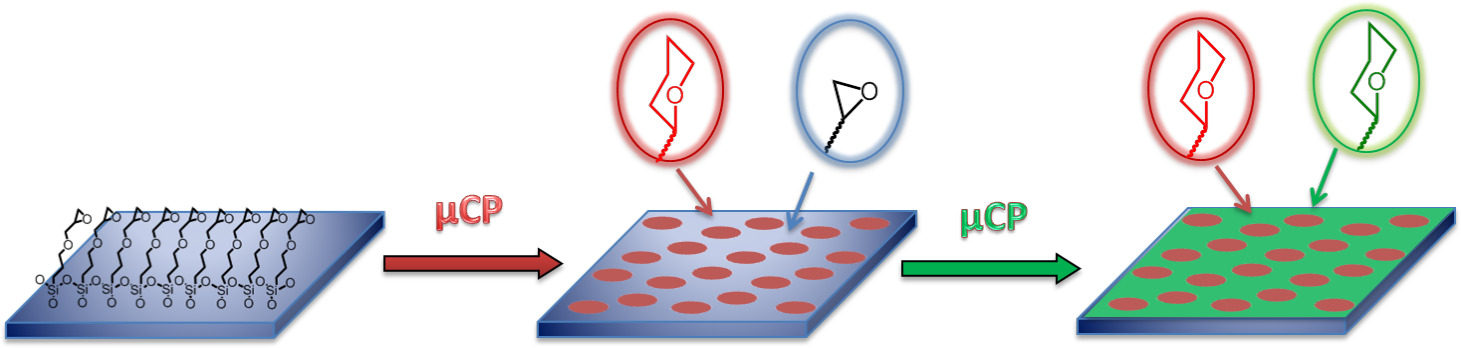
Schematic representation of the preparation of a simple carbohydrate microarray by μCP of amine-functionalized carbohydrate inks on epoxide-terminated SAM (“microcontact chemistry”). The first carbohydrate (red) is printed with a patterned PDMS stamp, the second carbohydrate (green) is printed in the interspaces with a flat PDMS stamp.

The µCP protocol to fabricate carbohydrate microarrays was optimized to provide a highly effective combination of stamp, ink and substrate. In view of the fact that the epoxide ring opening reaction with amines is much faster at elevated temperatures, the surface of oxidized, patterned PDMS stamps was coated with 2-[methoxy(polyethyleneoxy)propyl]trimethylsilane (PEG silane) [51]. The PEG coated stamps provided optimal wetting of the stamp by the carbohydrate ink solution, while preventing contamination of the substrate by PDMS residues. Moreover, it was observed that oxidized stamps without such a PEG coating adhered irreversibly to the epoxide substrates, possible due to reaction of the epoxide substrate with the oxidized PDMS surface. In the optimized µCP protocol, the PEG coated stamps were wetted with a 20 mM ethanolic solution of carbohydrate inks (NANA, Glc, Gal or Man) and triethylamine, and dried after 1 min incubation time. After placing the stamps on the epoxide-terminated SAMs on cleaned and activated glass or silicon substrates, the substrate and stamp were placed in an oven at 60 °C for 4 h. Finally, the stamp was removed from the substrate at room temperature. In order to prepare microarrays with two carbohydrates, the printing step was repeated with a second carbohydrate ink on a flat stamp to functionalize the remaining epoxide surface (Figure 2). After printing, the surfaces were rinsed extensively to remove any residual physisorbed material.

**Figure 3:**
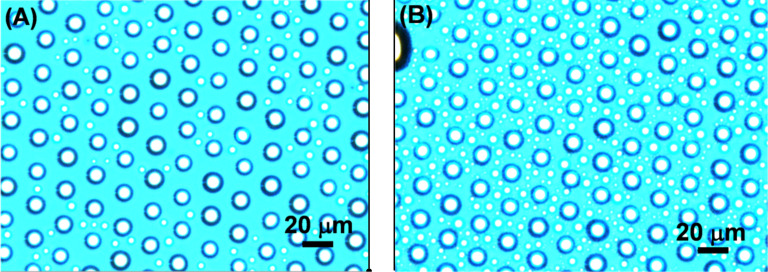
Optical microscopy images of water droplets selectively condensed in the areas where (A) the NANA ink and (B) the Gal ink were patterned by μCP on an epoxide-terminated SAM.

Immobilization of carbohydrates on the epoxide-terminated SAMs was investigated by several methods. Printing of a polar carbohydrate on the epoxide surface should lead to increased hydrophilicity exclusively in the printed areas which can be detected by water condensation experiments. Figure 3 shows optical microscopy pictures of epoxide SAMs onto which NANA ink or Gal ink were printed with a structured stamp (10 μm dots spaced by a 5 μm gap). It is obvious that the water condensed on the surface is found exclusively in the areas where the hydrophilic NANA or Gal were printed. Furthermore, quantification of the surface hydrophilicity was performed by water contact angle measurements. To this end, a flat PDMS stamp was used to obtain a homogenous carbohydrate surface after μCP for 4 h at 60 °C. After printing, the contact angles decreased from 61°/33° (advancing/receding) for the epoxide-terminated SAMs to ~30°/~12° for the carbohydrate-functionalized SAMs (with minor differences for the different carbohydrate inks). Data for all contact angle measurements can be found in Table S1 in Supporting Information File 1.

Further information on the printing process was obtained from X-ray photoelectron spectroscopy (XPS) of Si-wafers functionalized with an epoxide-terminated SAM and printed with NANA ink using a PEG coated flat stamp (see Figure S3 and Figure S4 in Supporting Information File 1). While nearly no N(1s) signal was detected in the epoxide-terminated SAM, a clear N(1s) signal can be observed when the NANA ink is printed. The C(1s) peak shows a splitting into the C–C (285 eV), C–O (287 eV), C=C, C=O and residual epoxide C–O signals (289 eV) as expected for the NANA-terminated SAM [52].

**Figure 4:**
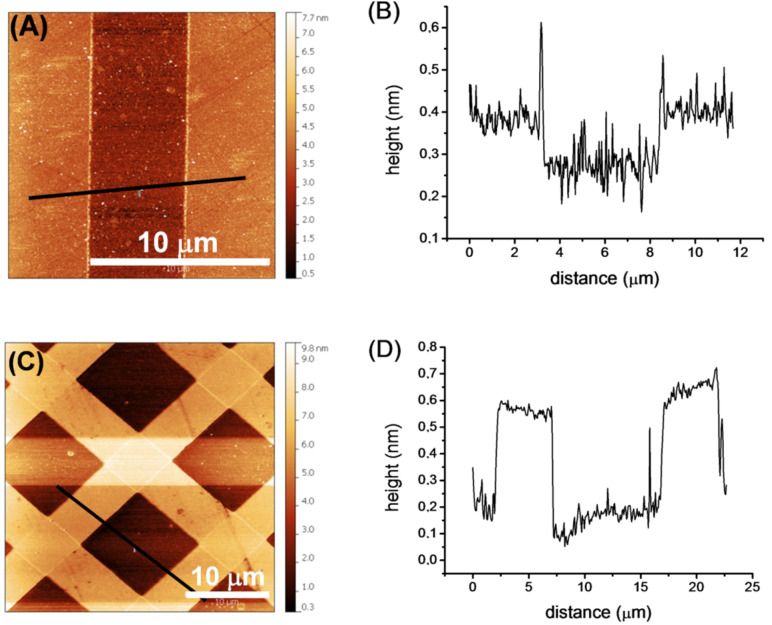
(A) AFM height image (zoom) of NANA ink in 10 μm stripes on an epoxide-terminated SAM; (B) Height profile of the black line shown in (A); (C) AFM height image of Glc ink and Gal ink cross printed in 5 μm stripes on an epoxide-terminated SAM; (D) Height profile of the black line shown in (C).

In addition, atomic force microscopy (AFM) of patterned epoxide SAMs was performed to verify the success of the printing process. The AFM height profiles of a pattern of NANA ink printed with a patterned stamp (10 μm stripes that are spaced by a 5 μm gap) as well as a cross printed pattern obtained by a successive print at 90° angle with two identically patterned stamps (5 μm stripes that are spaced by a 10 μm gap) using the Glc ink and the Gal ink are shown in Figure 4. A clear pattern in accordance with the shape and dimensions of the used stamps can be seen. If printing of the carbohydrate ink results in a very dense layer of carbohydrates aligned mostly perpendicular to the surface, the height difference of printed and nonprinted areas should be around 1 nm. The observed height differences are significantly less which indicates that the carbohydrates are tilted relative to the surface normal and that the printed layers exhibit a lower than maximal surface coverage. This is most likely due to a combination of factors, i.e., quality of the base epoxide SAM, reaction time with the amine inks, and steric bulk of the carbohydrates compared to the triethylene glycol linker. We note that these observations are consistent with our earlier reports on the immobilization of glycosides on SAMs by µCP [33–36].

**Figure 5:**
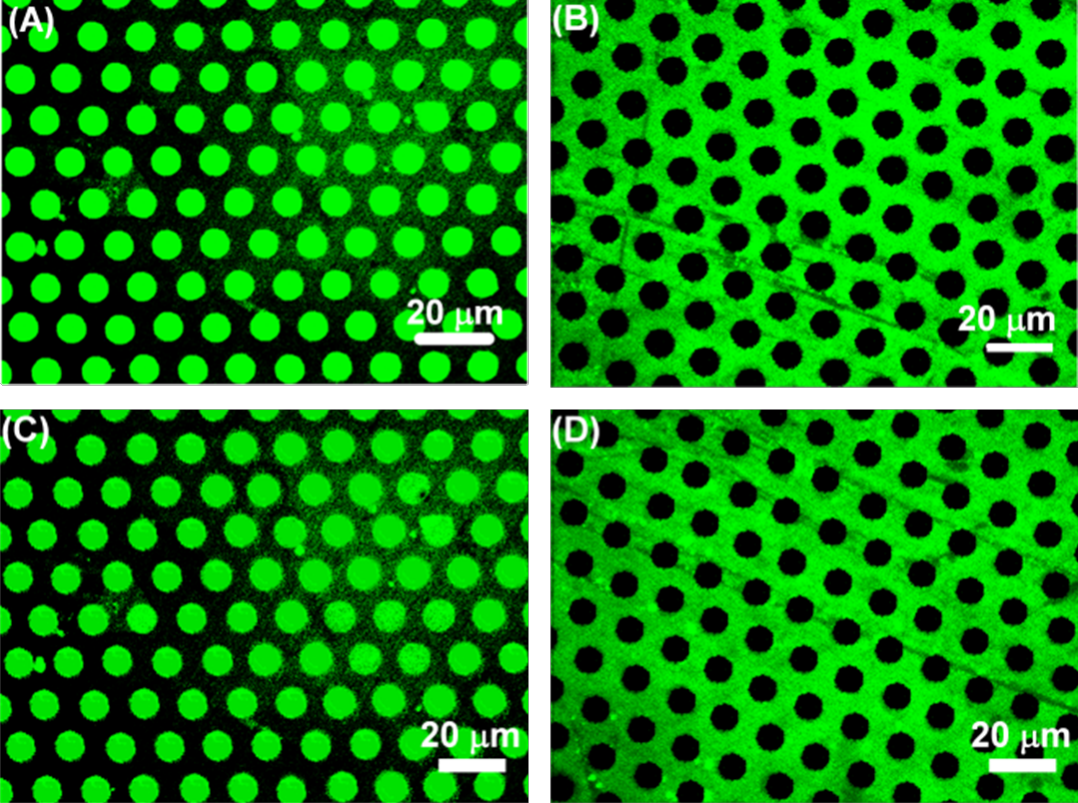
Fluorescence images of bifunctional carbohydrate microarrays incubated with FITC-HisHis. (A) NANA (dots 10 × 5 μm) and Man (background); (B) Man (dots 10 × 5 μm) and NANA (background); (C) Gal (dots 10 × 5 μm) and Man (background); (D) Man (dots 10 × 5 μm) and Gal (background).

Having established that one or more carbohydrate inks can be patterned on epoxide-terminated SAMs by µCP, we turned our attention to the investigation of the interaction of synthetic and natural lectins with the immobilized carbohydrates. To this end, bifunctional surfaces were fabricated by µCP of a the first ink in a dot pattern (10 μm dots spaced by a 5 μm gap) and filling up the interspaces with the second ink by using µCP with a flat stamp. In a first set of experiments, NANA ink was printed in a dot pattern and the remaining area was functionalized by printing Man ink with a flat stamp. In a reverse experiment, Man ink was printed in dots and the remaining area was functionalized with NANA ink. After incubation of these carbohydrate arrays with the synthetic lectin FITC-HisHis, fluorescence was observed exclusively in the areas where the NANA ink had been printed. No significant fluorescence was observed in the area where the Man ink had been printed (Figure 5). This observation confirms – for the first time – that the synthetic lectin HisHis binds selectively to NANA, and not to Man, also when the carbohydrate is immobilized on a surface. We note that no fluorescence was detected when the inks were printed onto bare glass slides (instead of epoxide-functionalized glass slides) or when printing was performed without inking the PEG coated PDMS stamp. In a second set of experiments, NANA ink was printed in dots and the remaining area was functionalized with Glc ink, and vice versa. Again it was observed that HisHis binds exclusively to NANA, and not to Glc (see Figure 8A). In a third set of experiments, Gal ink was printed in dots and the remaining area was functionalized with Man ink, and vice versa. As expected, it was observed that FITC-HisHis binds to Gal, and not to Man (Figure 5). In our preceding work, we had observed that the parallel isomer of HisHis binds to β-galactosides [40].

In order to confirm the interaction between HisHis and NANA with surface plasmon resonance (SPR) spectroscopy a commercially available polycarboxylate hydrogel sensor surface was employed, which is known to be particularly advantageous for low molecular weight compounds due to the signal amplification caused by multiple binding events in the hydrogel on the sensor. The functionalization of the polycarboxylate hydrogel with amine terminated NANA was performed by *N*-hydroxysuccinimide (NHS) activation and subsequent peptide coupling. Indeed, using the hydrogel sensor a small but significant SPR signal increase was observed upon the addition of HisHis (see Figure S5 in Supporting Information File 1). The initial rate and extent of surface binding correlated with the concentration of HisHis (0.5–2.0 mM) applied to the sensor. However, it was not possible to obtain sufficiently reproducible data to perform a quantitative analysis of the peptide–carbohydrate interaction. The poor quality of the SPR signal is certainly due to the low molecular weight of HisHis which limits any further SPR investigations.

**Figure 6:**
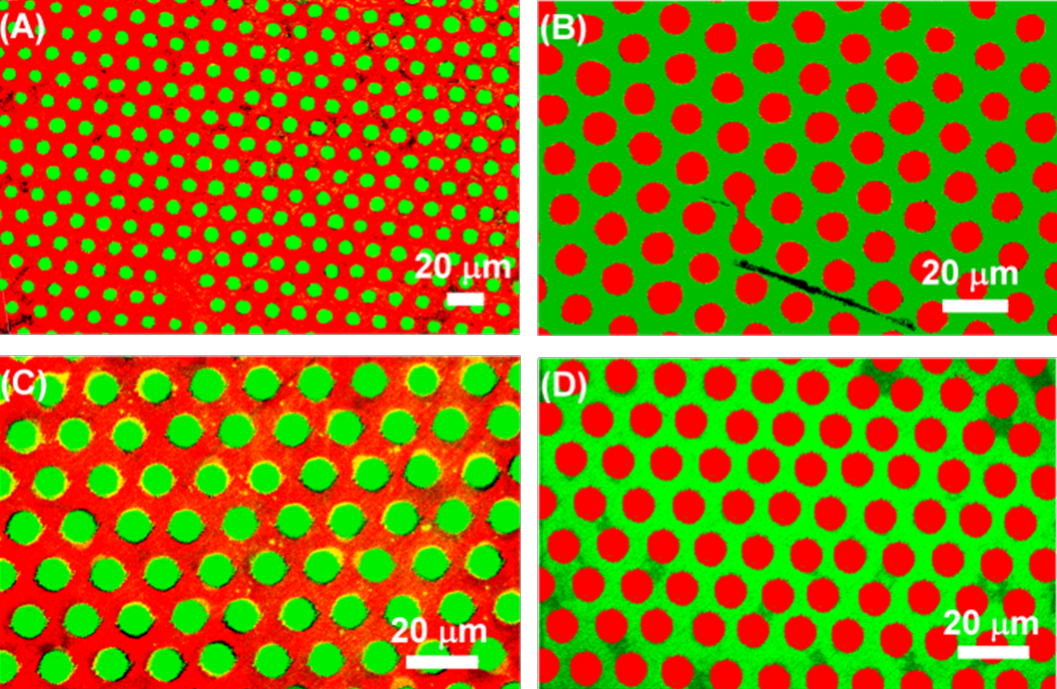
Overlay of fluorescence images of bifunctional carbohydrate microarrays; (A) NANA (dots 10 × 5 μm) and Man (background) incubated with FITC-HisHis and TRITC-ConA; (B) Man (dots 10 × 5 μm) and NANA (background) incubated with FITC-HisHis and TRITC-ConA; (C) Gal (dots 10 × 5 μm) and Man (background) incubated with FITC-HisHis and TRITC-ConA; (D) Man (dots 10 × 5 μm) and Gal (background) incubated with FITC-PNA and TRITC-ConA.

Additionally, bifunctionalized carbohydrate surfaces were incubated simultaneously with FITC-HisHis, TRITC-ConA, and FITC-PNA to elucidate whether selective binding of the synthetic lectin to glycosides is also observed in the presence of natural lectins. In Figure 6, the overlays of fluorescence images are shown. In each experiment, FITC-HisHis is detected exclusively on the NANA and the Gal areas, whereas TRITC-ConA binds to Man and FITC-PNA binds to Gal only. These observations demonstrate that selective recognition between HisHis and NANA or Gal is not in any way disturbed by the presence of a second lectin and that the synthetic lectin HisHis and the natural lectins ConA and PNA operate orthogonally.

**Figure 7:**
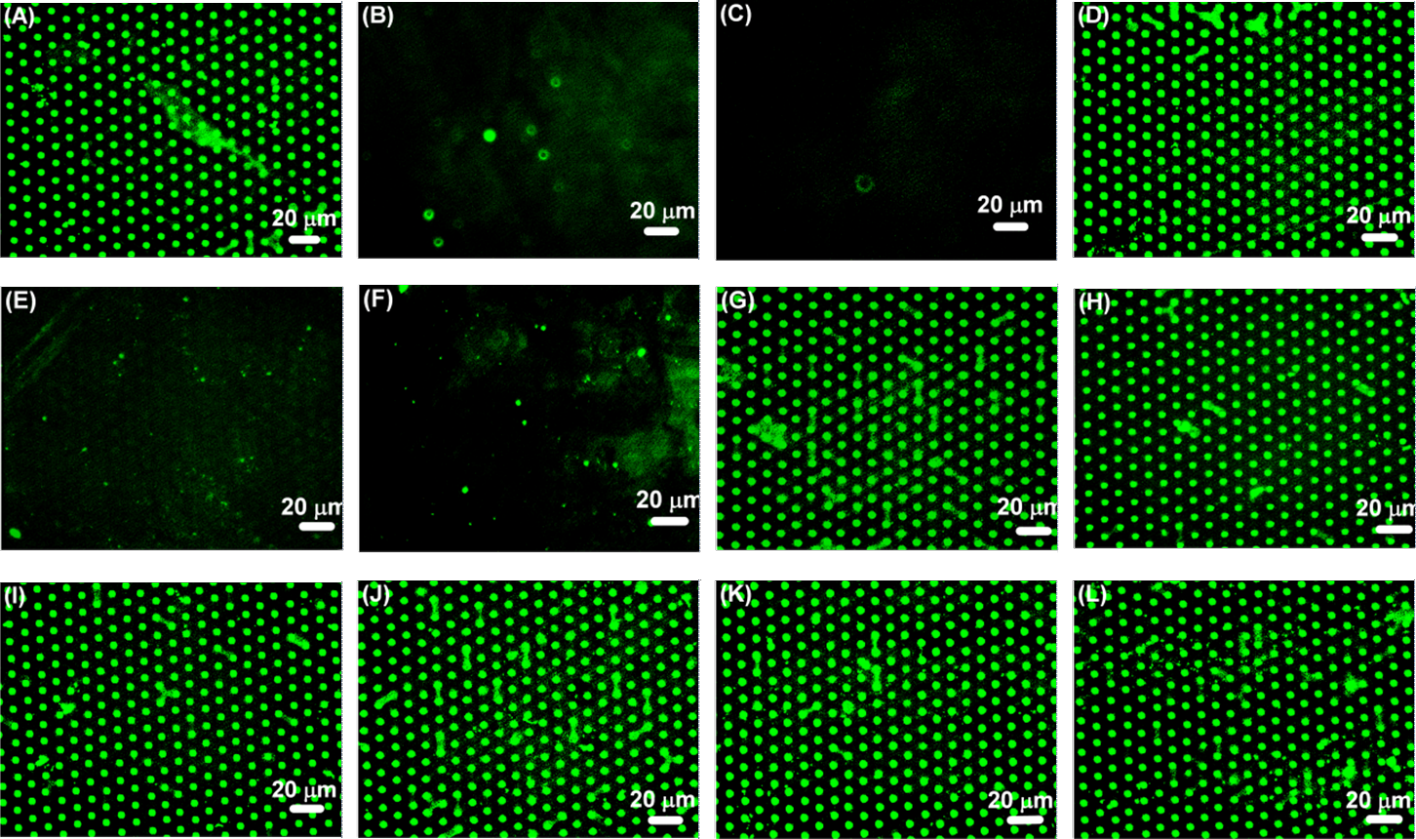
Fluorescence images of a microarray consisting of NANA (dots 5 × 3 μm) and Man (background). (A) Incubation with FITC-HisHis; (B) washing of (A) with 250 mM NANA; (C) washing of (A) with 500 mM methyl β-D-galactoside; (D) incubation of (B) with FITC-HisHis; (E) simultaneous incubation with FITC-HisHis and NANA; (F) simultaneous incubation with FITC-HisHis and methyl β-D-galactoside; (G) washing of (A) with 500 mM methyl α-D-mannoside; (H) washing of (A) with 500 mM methyl β-D-glucoside; (I) washing of (A) with 500 mM methyl β-D-fucoside; (J) washing of (A) with 500 mM trehalose; (K) washing of (A) with 500 mM sucrose; (L) washing of (A) with 500 mM *N-*acetylglucosamine.

Additionally, the selectivity of the FITC-HisHis was tested with a set of competition experiments on a microarray of NANA dots with Man background (Figure 7). If the recognition process between FITC-HisHis and NANA or Gal is selective, incubation with a large excess of the two carbohydrates should lead to release of the surface-bound synthetic lectin from a microarray displaying NANA or Gal. In this case, the detected fluorescence pattern should vanish after washing the microarrays with concentrated solutions of NANA and Gal. As is shown in Figure 7B,C, FITC-HisHis is indeed displaced from the microarray when a solution of 250 mM NANA or a solution of 500 mM methyl β-D-galactoside solution is used to rinse the microarray. The original fluorescence pattern can be restored by incubation with FITC-HisHis (Figure 7D) and this process is reversible over several cycles. Upon simultaneous incubation of the microarray with NANA or methyl β-D-galactoside and FITC-HisHis, no fluorescent pattern was detected (Figure 7E,F). This indicates that the synthetic lectin HisHis is saturated by the excess of carbohydrate which is present in solution and can therefore no longer bind to the carbohydrates on the microarray. Conversely, it would be expected that carbohydrates which do not show any interaction with HisHis should not lead to a decrease in the fluorescence of FITC-HisHis on the microarray and this is indeed observed for methyl α-D-mannoside, methyl β-D-glucoside, methyl β-D-fucoside, trehalose, sucrose as well as *N-*acetylglucosamine (see Figure 7G–L). These experiments demonstrate two important points: firstly, they attest the selectivity as well as the affinity of HisHis for immobilized NANA and Gal, and secondly, these results show that even a very simple microarray provides a versatile screening tool.

**Figure 8:**
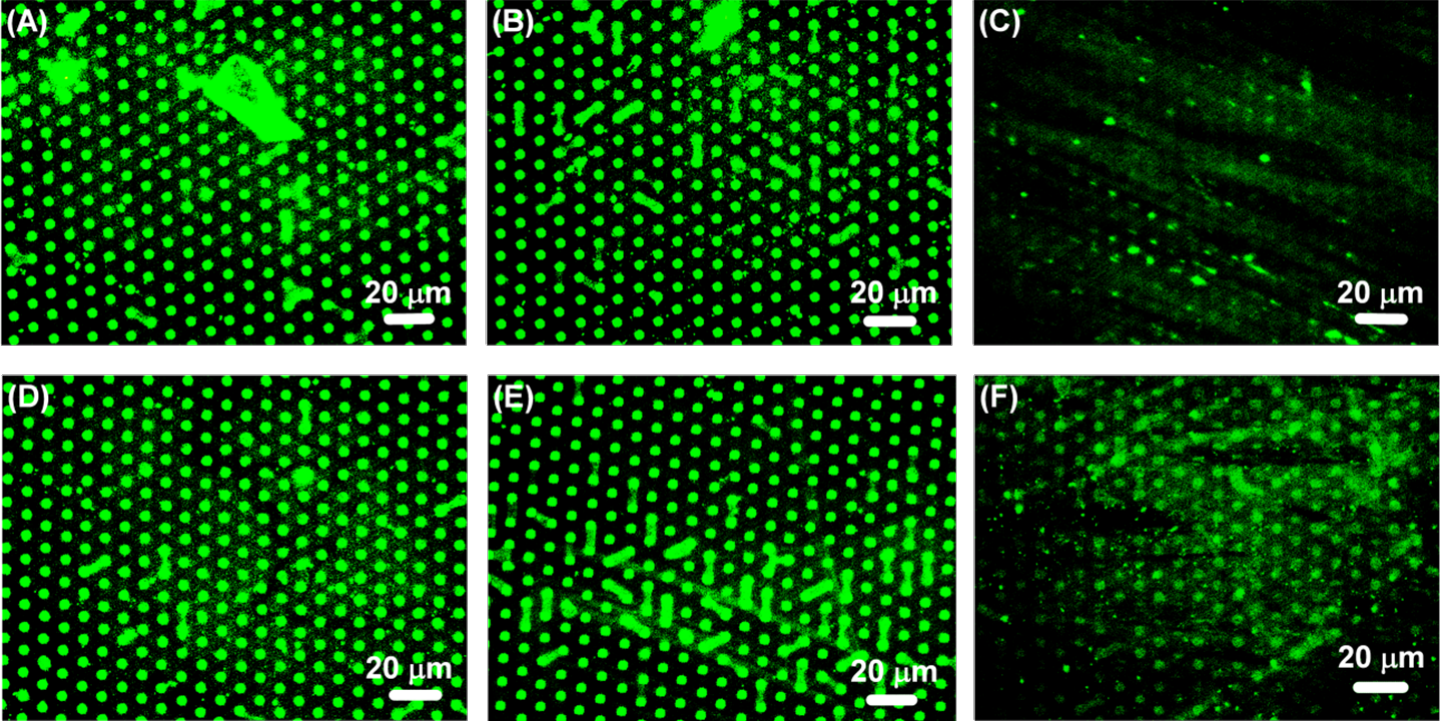
Fluorescence images of a microarray of NANA (dots 5 × 3 μm) and Glc (background), first incubated with FITC-HisHis and subsequently incubated for 5 h with (A) a 1000-fold dilution of KO (0.00612 µg µL^−1^); (B) a 100-fold dilution of KO (0.0612 µg µL^−1^); (C) a 10-fold dilution of KO (0.612 µg µL^−1^); (D) a 1000-fold dilution of WT (0.00661 µg µL^−1^); (E) a 100-fold dilution of WT (0.0661 µg µL^−1^); (F) a 10-fold dilution of WT (0.661 µg µL^−1^).

Finally, in addition to the mono- and disaccharides described above, the selectivity of the synthetic lectin HisHis was tested by using two heparins. These polymers display a particularly high carbohydrate epitope density. While one polymer (wild type, WT, 6.61 µg µL^−1^) has a negatively charged sulfonate group at the terminus, the second one (KO, 6.12 µg µL^−1^) does not possess this group and is therefore charge neutral. After 5 h incubation of a microarray of NANA (10 μm dots spaced by a 5 μm gap) and Glc (background) with three different dilutions of WT and KO, the fluorescence images were recorded (Figure 8). No significant effect is observed when the polysaccharides are diluted by a factor of 1000 or a factor of 100, but a 10 times dilution of KO and WT (0.661 µg µL^−1^ and 0.612 µg µL^−1^) leads to a strong decrease of the FITC-HisHis fluorescence on the microarray (Figure 8C,F). No loss in fluorescence was detected when the wafer was incubated for 5 h with the same amount of Milli-Q water. These data demonstrate that heparin-type polysaccharides can compete with the interaction of the synthetic lectin HisHis and the carbohydrate NANA. The concentration dependence of displacement assay indicates the high affinity of HisHis for NANA in the microarray. Thus, the results obtained from the microarray support our earlier finding that the interaction of HisHis and NANA is 1:2 [39], so that HisHis is likely to bind divalently to the microarray surface.

## Conclusion

In summary, we have investigated the molecular recognition of surface immobilized carbohydrates by a synthetic lectin. To this end, amine-tethered carbohydrates were printed on epoxide SAMs by μCP. Using tailor-made PDMS stamps and an optimized printing protocol, simple carbohydrate microarrays could readily be obtained by μCP. Consistent with measurements in solution, the microarrays showed high selectivity of the synthetic lectin HisHis for NANA and Gal versus Glc and Man. Both the selectivity and the high affinity of the synthetic lectin could be demonstrated in competition experiments with monosaccharides, disaccharides and heparin-type polysaccharides. The results obtained here demonstrate – for the first time – the high selectivity and affinity of a synthetic lectin for two different carbohydrates immobilized in a simple microarray. We contend that this is a significant development in the search for synthetic lectins that operate under physiological conditions.

## Experimental

**General:** Chemicals were purchased from Sigma Aldrich, Acros Organics, Iris Biotech and ABCR and used without further purification. Silicon wafers (B-doped, 100-orientation, resistivity 20–30 Ω) were kindly donated by Siltronic AG. The heparins KO and WT were kindly donated by Prof. Dr. Rupert Hallmann (Westfälische Wilhelms-Universität Münster). Milli-Q water was prepared from distilled water using a PureLab UHQ deionization system (Elga). Tetramethylrhodamine isothiocyanate-labeled concanavalin A (TRITC-ConA) was purchased from Sigma Aldrich. Fluorescein isothiocyanate-labeled peanut agglutinin (FITC-PNA) was obtained from Vector Laboratories. Detailed information concerning the synthesis and analysis of carbohydrates and peptides is provided in Supporting Information File 1.

**SAM preparation:** Epoxide-terminated SAMs were prepared as reported in literature [42]. Glass slides as well as Si-wafers were cut into small pieces of around 1.6 × 2.6 cm^2^, cleaned with detergent and dried. After sonification in pentane, acetone and Milli-Q water, the slides were put into a freshly prepared piranha solution (H_2_O_2_/H_2_SO_4_ = 1:3) for 30 min. The wafers were then thoroughly washed with water, dried and immersed in a 0.3 vol % solution of (3-glycidoxypropyl)trimethoxysilane in toluene for 24 h. Excess silane was removed by sonification in abs. ethanol for 20 min. After washing the slides with abs. ethanol, and drying, they were kept at least 24 h under ambient conditions before printing.

**Stamp preparation:** Poly(dimethylsiloxane) (PDMS) stamps were prepared by mixing PDMS with a curing agent (Sylgard 184, Dow Corning) in a 10:1 ratio and pouring the viscous mixture on a patterned silicon master. After removing the air in vacuum, the PDMS was cured at 80 °C overnight and peeled off after cooling. The patterned areas were cut out with a knife and oxidized in a UV-ozonizer (PSD-UV, Novascan Technologies Inc.) for 55 min after which they were stored under water. Coating of the stamps with 2-[methoxy(poly(ethyleneoxy))propyl]trimethylsilane was done by incubating the oxidized and dried stamps in a 1 vol % solution of the silane in abs. ethanol for 2 h, after which they were washed with abs. ethanol and dried [51]. Flat PDMS stamps were fabricated as described above by using a flat silicon wafer as master.

**Microcontact printing:** The surface of the freshly oxidized or coated PDMS stamp was covered with 2 to 3 drops of ethanolic solutions of the ink (20 mM) and triethylamine (20 mM) and incubated for 1 min. After blow-drying, they were placed on the according SAM. Printing was performed by placing the substrates together with the stamp in an oven which was tempered to 60 °C for 4 h. After removing the stamp from the substrate, with dichloromethane (DCM), abs. ethanol and Milli-Q water and dried. Subsequent printing steps were carried out as described above. After the last print, the substrates were sonicated in DCM, abs. ethanol and Milli-Q water, and dried.

**Lectin carbohydrate interactions:** As described in [34], in order to reduce non-specific protein adsorption, the arrays were incubated with a 3% bovine serum albumin (BSA) solution in PBS buffer (pH 7.5, 0.1% Tween 20) for 30 min and washed two times with PBS buffer prior to lectin screening. The surfaces of the carbohydrate arrays were covered by a solution of 1 mM FITC HisHis and 1 μg of labeled lectin (TRITC ConA or FITC PNA) in 100 μL of HEPES buffer (20 mM HEPES, pH 7.5, 0.15 M NaCl, 1.0 mM CaCl_2_). In the case of ConA, MnCl_2_ was added to a concentration of 1 mM. After 90 min, the arrays were washed with the same buffer, rinsed with Milli-Q water, dried, and analyzed.

**Selectivity studies:** The carbohydrate chips were incubated with a 1 mM solution of FITC HisHis for 1 min, washed with phosphate buffer (100 mM, pH 7.4) to remove unbound receptor, dried and analyzed. Afterwards, the wafer was washed three times with 500 μL mono- or disaccharide solution, dried and analyzed again. 100 μL of the heparin polymer (KO and WT) solutions were put on glass slides which were afterwards covered with a beaker. After 5 h at room temperature, the glass slides were washed with Milli-Q water, dried and analyzed.

**Contact angle measurements**: Water contact angles were measured using the sessile drop method on a DSA 100 goniometer (Krüss GmbH Wissenschaftliche Laborgeräte, Hamburg/Germany). The advancing and receding contact angles were measured on glass and silicon substrates, and at least three measurements were performed for every sample. Determination of the angles was done using the software Drop Shape Analysis.

**X-ray photoelectron spectroscopy (XPS):** X-ray photoelectron spectra were recorded on a Kratos Axis Ultra system (Kratos Analytical, Manchester/UK). Monochromatized Al Ka radiation (1486.6 eV) as the excitation source and a pass energy of 20 meV for narrow scans were used. The obtained spectra were analyzed using the Casa XPS (version 2.3.15, Casa software Ltd, Teignmouth/UK) software and were referenced to the C(1s)-peak of the saturated hydrocarbons by setting it to 285 eV. All measurements were carried out on silicon substrates.

**Atomic force microscopy (AFM):** AFM images were measured with a Nano Wizzard 3 system (JPK Instruments AG, Berlin/Germany) in combination with processing software Gwyddion (http://www.gwyddion.net, version 2.25). All measurements were carried out on silicon substrates.

**Fluorescence microscopy:** Fluorescence microscopy images were made by using an Olympus inverted research microscope CKX41 (Olympus, Shinjuku, Tokyo/Japan) equipped with a mercury burner U-RFL-T as light source and a DX 20 L-FW camera (Kappa opto-electronics GmbH, Gleichen/Germany) for image acquisition. The camera was controlled by the program Kappa CameraControl (version 2.7.5.7032). All experiments were carried out on glass substrates.

## Supporting Information

File 1 Experimental procedures, characterization data and additional spectra.
