# Supplementary material for: Molecular recognition of surface-immobilized carbohydrates by a synthetic lectin
Source: Beilstein J Org Chem. 2014 Jun 16;10:1354–64. doi: 10.3762/bjoc.10.138 (PMC4077543; doi:10.3762/bjoc.10.138)
Supplement: File 1 — Experimental procedures, characterization data and additional spectra. [file Beilstein_J_Org_Chem-10-1354-s001.pdf]

Supporting Information

for

Molecular recognition of surface-immobilized carbohydrates by a synthetic lectin

Melanie Rauschenberg, Eva-Corrina Fritz, Christian Schulz, Tobias Kaufmann and Bart Jan Ravoo*

Address: Organic Chemistry Institute, Westfälische Wilhelms-Universität Münster, Corrensstrasse 40, 48149 Münster, Germany

Email: Bart Jan Ravoo - b.j.ravoo@uni-muenster.de

*Corresponding author

Experimental procedures, characterization data and additional spectra

Synthesis

General

Chemical were purchased from Sigma Aldrich, Acros Organics, Iris Biotech and used without further purification. Methanol and dimethylformamide (DMF) were dried by storage over molecular sieves (3 Å). Dichloromethane (DCM) was dried by distillation over CaH_2 under argon. Tetrahydrofuran was dried by distillation over potassium/benzophenone under argon. For peptide coupling reactions, DMF SPPS grade was used. Reactions in solution were monitored by thin-layer chromatography (TLC) performed on 0.2 mm Merck precoated silica gel 60 F_{254} aluminium sheets. Spots were visualized by dipping into basic $KMnO_4$ solution. Silica gel column chromatography was carried out on silica gel 60 (0.063–0.20 mm, Merck). Coupling and deprotection steps in the SPPS were monitored with the Kaiser test. NMR spectra were recorded on Bruker spectrometers (AV300, AV400). Chemical shifts are relative to residual signals of the deuterated solvents and are reported in units of parts per million (ppm). Coupling constants (*J*) are given in Hertz (Hz). Mass spectra were recorded on a MicroToF spectrometer (Bruker).

Strategy

2-{2-[2-(Benzyloxycarbonylamino)ethoxy]ethoxy}ethanol **3** was synthesized starting from 2-(2(2-chloroethoxy)ethoxy)ethanol in a three step synthesis (Scheme S1).

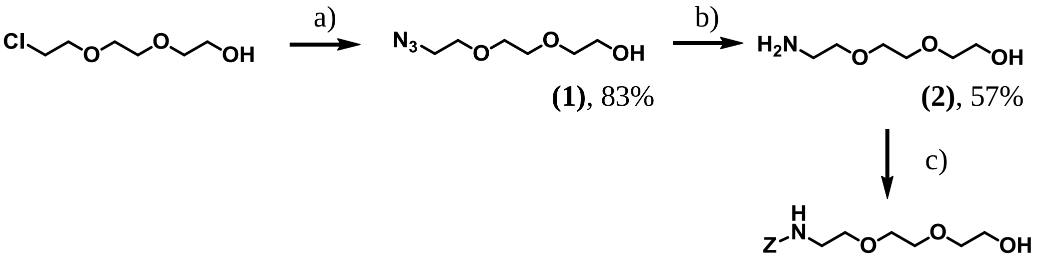

(**1**), 83%

(**2**), 57%

(**3**), 60%

Scheme S1: Synthesis of the spacer (3); a) NaN_3, DMF, 24 h, 90 °C; b) PPh_3, THF, H_2O, 48 h, rt; c) Z-Cl, NEt_3, DCM, 24 h, rt.

1-Amino-8-(benzyloxycarbonylamino)-3,6-dioxaoctane (**4**) was prepared by protection of 1,8-diamino-3,6-dioxaoctane with Z-chloride (Scheme S2).

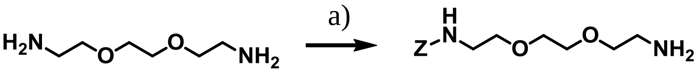

(**4**), 87%

Scheme S2: Synthesis of spacer (4); a) Z-Cl, NEt_3, DCM, 24 h, rt.

The β-D-glucopyranosyl-amino-conjugate **7** (Glc) and the β-D-galactopyranosyl-amino-conjugate **10** (Gal) were synthesized starting from peracetylated β-D-glucose and β-D-galactose in a three step synthesis (Scheme S3) while a four step synthesis was used for the β-D-mannopyranosyl-amino-conjugate **14** (Man) starting from peracetylated β-D-mannose (Scheme S4).

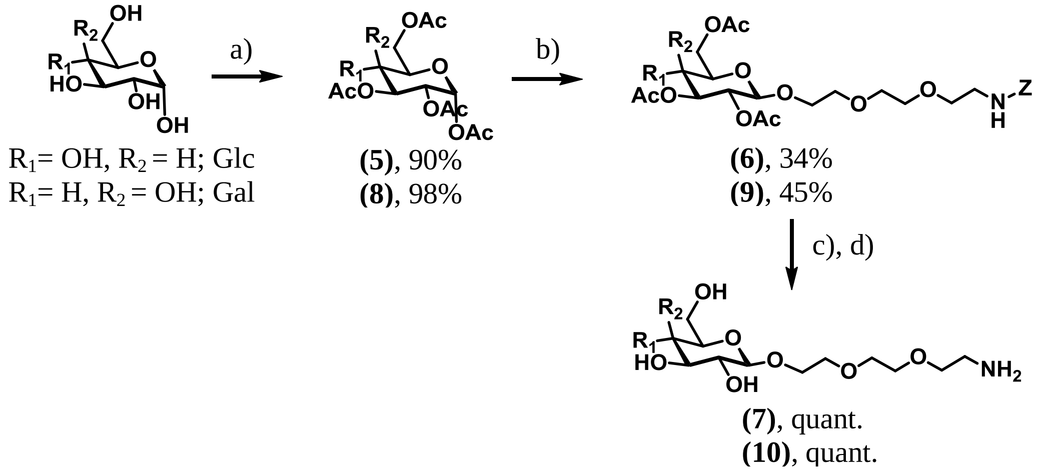

Scheme S3: Synthesis of the β-D-glucopyranosyl-amino-conjugate 7 (Glc) and β-D-galactopyranosyl-amino-conjugate 10 (Gal); a) NaOAc, Ac_2O, 30 min, reflux; b) $BF_3{\cdot}(OEt)_2$, 3, DCM, 24 h, rt; c) cat. NaOMe, MeOH, 1 h, rt; d) H_2, Pd/C, MeOH, 24 h, rt.

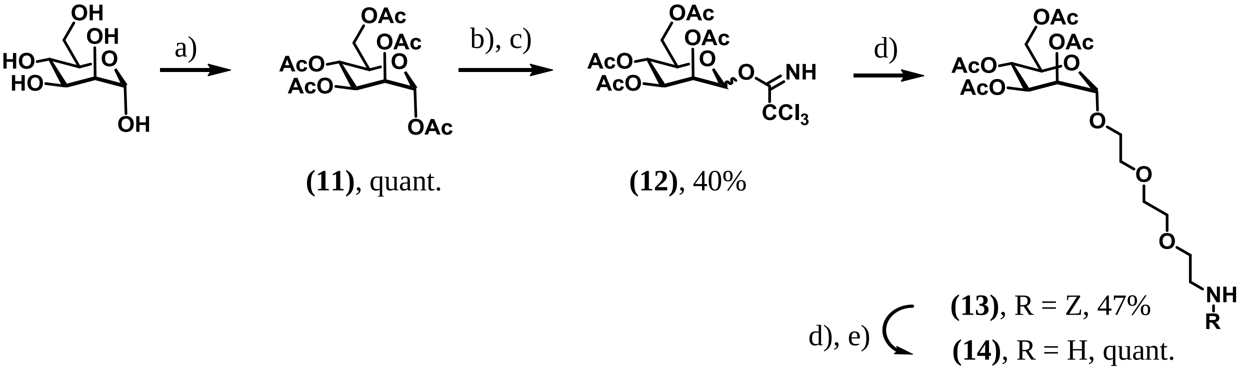

Scheme S4: Synthesis of the mannose ink 14 (Man); a) NaOAc, Ac_2O, reflux, 30 min; b) hydrazine acetate, DMF, 1 h, 60 °C; c) trichloroacetimidate, DBU, DCM, 1 h, 0 °C, 1 h, rt; d) 3, TMSOTf, 4 Å MS, DCM, 24 h, −30 °C → rt; e) cat. NaOMe, MeOH, 1 h, rt; f) H_2, Pd/C, MeOH, 24 h, rt.

The amine-tethered *N*-acetyl neuraminic acid **16** (NANA) was synthesized starting from *N*-acetylneuraminic acid (Scheme S5).

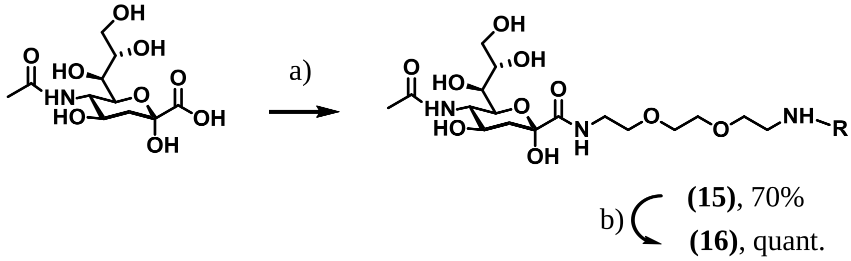

Scheme S5: Synthesis of the amine-functionalized *N*-acetylneuraminic acid 16 (NANA); a) 4, Oxyma pure®, DIPCDI, DCM, 24 h, rt; b) H_2, Pd/C, MeOH, 24 h, rt.

The synthesis of the fluorescently labeled synthetic lectin **22** (FITC-HisHis) was achieved using SPPS according to Scheme S6.

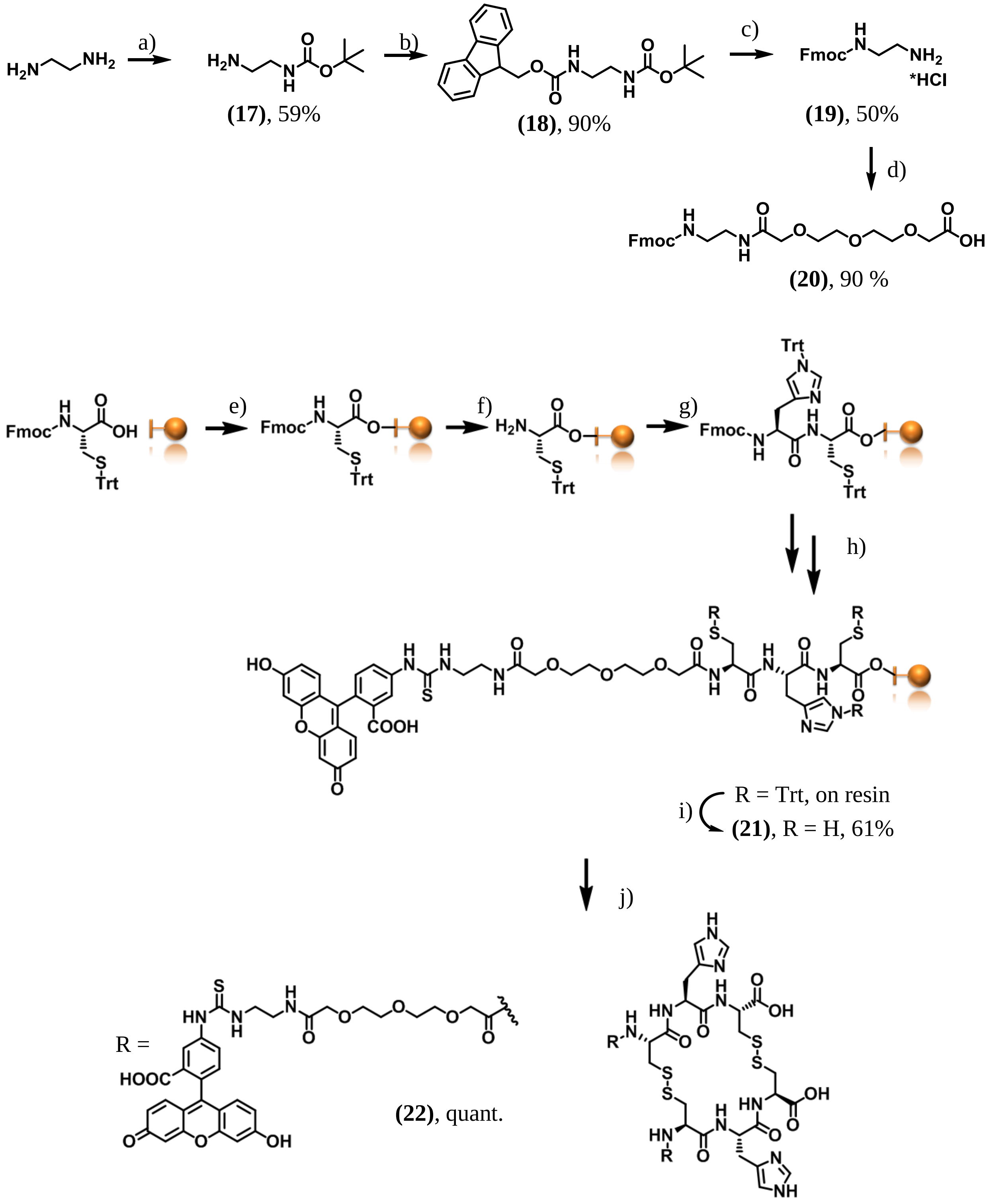

Scheme S6: Synthesis of the fluorescently labeled synthetic lectin 22 (FITC-HisHis); a) Boc_2O, DCM, 24 h, 0 °C → rt; b) Fmoc-OSu, H_2O/ACN, $NaHCO_3$, 4 h, rt; c) conc. HCl, EtOAc, 4 h, rt; d) 3,6,9-trioxaundecanedioic acid, Oxyma pure®, DIPCDI, NMM, DCM, 24 h, rt; e) DCM, DMF, DIPEA, 1 h, rt, MeOH, 15 min, rt; f) 20% piperidine in DMF, 20 min, rt; g) Fmoc-His(Trt)-OH, Oxyma pure®, DIPCDI, DMF, 1 h, rt; h) 20% piperidine in DMF, 20 min, rt; Fmoc-Cys(Trt)-OH, Oxyma pure®, DIPCDI, DMF, 1 h, rt; 20% piperidine in DMF, 20 min, rt; 20, Oxyma pure®, DIPCDI, DMF, 24 h, rt; 20% piperidine in DMF, 20 min, rt; fluorescein isothiocyanate, DIPEA, DMF, 24 h, rt; i) 94% TFA, 2.5% H_2O, 2.5% EDT, 1% TIS, 6 h, rt; j) 100 mM phosphate buffer, 48 h, rt.

2-[2-(2-Azidoethoxy)ethoxy]ethanol (1)

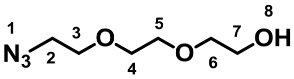

The synthesis was performed according to literature.[1] A mixture of 2-(2-(2-chloroethoxy)ethoxy)ethanol (6.00 mL, 41.5 mmol) and sodium azide (2.70 g, 41.5 mmol) in dry DMF p.a. (45 mL) was stirred at 90 °C overnight. After cooling the solution was diluted with THF (50 mL) and stirred for 1 h at room temperature. The crude product was obtained after filtration, washing of the solid with THF (200 mL) and removal of the solvent under reduced pressure.

Molecular formula: $C_6H_{13}N_3O_3$ (yellowish oil).

Yield: 6.00 g (34.3 mmol, 83%).

^{1}H NMR (300 MHz, $CDCl_3$, 298 K): δ = 3.70 – 3.58 (m, 8H, 2,4,5,7-H), 3.57 – 3.51 (m, 2H, 6-H), 3.37 – 3.30 (m, 2H, 3-H) ppm.

^{13}C NMR (75 MHz, $CDCl_3$, 298 K): δ = 72.5 (6-C), 70.5 (4,5-C), 69.9 (3-C), 61.5 (8-C), 50.6 (2-C) ppm.

2-[2-(2-Aminoethoxy)ethoxy]ethanol (2)

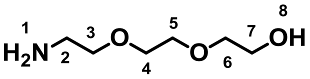

The synthesis was done according to literature under argon.[1] The crude educt **1** (6.00 g, 34.4 mmol) was dissolved in THF (50 mL) and triphenylphosphine (11.7 g, 44.7 mmol) was added in two portions. After stirring for 48 h at room temperature, water (1.13 mL) was added and stirring was continued for another 48 h. The solvent was removed under reduced pressure and the residue is afterwards dried under high vacuum at 45 °C overnight. Water (119 mL) was added and the formed precipitate is removed by filtration. After washing the solid with water (200 mL), the solvent was evaporated under reduced pressure and the product was dried in high vacuum at 70 °C overnight.

Molecular formula: $C_6H_{15}NO_3$ (colorless oil).

Yield: 4.63 g (31.0 mmol, 90%).

ESI-HRMS (MeOH) (m/z): Calculated for $[C_6H_{15}NO_3H]^+$: 150.1125; found: 150.1127.

^{1}H NMR (300 MHz, DMSO-d_6, 298 K): δ = 3.31 – 3.03 (m, 10H, 2,4,5,6,7-H), 2.59 (br-s, 2H-1-NH_2), 2.37 (t, J = 5.8 Hz, 2H, 3-H) ppm.

^{13}C NMR (75 MHz, DMSO-d_6, 298 K): δ = 73.0 (6-C), 69.7 (4,5-C), 69.6 (3-C), 60.1 (8-C), 41.3 (2-C) ppm.

[1] Tabatadze, D.; Zamecnik, P.; Yanachkov, I.; Wright, G.; Pierson, K.; Zhang, S.; Bogdanov, A. Jr.; Metelev, V.; *Nucleosides, Nucleotides, and Nucleic Acids* **2008**, *27*, 157.

2-{2-[2-(Benzyloxycarbonylamino)ethoxy]ethoxy}ethanol (3)

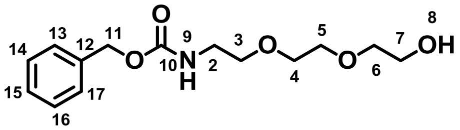

The synthesis was done according to literature under argon.[2] A solution of **2** (3.00 g, 20.1 mmol) in water (44 mL) was cooled to 0 °C. In five portions, $NaHCO_3$ (1.71 g, 20.3 mmol) and Z-chloride (3.55 mL, 26.1 mmol) in toluene (2.3 mL) were added and the solution was stirred overnight at room temperature. After separation of the layers, the aqueous layer was extracted with EtOAc (3 × 50 mL) and the organic phase was washed with brine (1 × 50 mL). Drying with $MgSO_4$, filtering and evaporating the solvent under reduced pressure gave the crude product, which was purified by silica gel column chromatography using EtOAc as eluent.

Molecular formula: $C_{14}H_{21}NO_5$ (colorless oil).

Yield: 1.66 g (5.87 mmol, 29%).

R_f value (EtOAc): 0.82.

ESI-HRMS (MeOH) (m/z): Calculated for $[C_{14}H_{21}NO_5Na]^+$: 306.1312; found: 306.1305.

^{1}H NMR (400 MHz, $CDCl_3$, 298 K): δ = 7.36 – 7.01 (m, 5H, 13,14,15,16,17-H), 5.42 (br-s, 1H, 9-NH), 5.03 (s, 2H, 11-H), 3.69 – 3.38 (m, 10H, 2,4,5,6,7-H), 3.34 (dt, *J* = 15.6, 5.1 Hz, 2H, 3-H), 2.63 (s, 1H, 8-H) ppm.

^{13}C NMR (101 MHz, $CDCl_3$, 298 K): δ = 155.5 (10-C), 135.6 (12-C), 131.1 (15-C), 127.5 (13,14,16,17-C), 71.5 (6-C), 69.3 (4,5-C), 69.1 (3-C), 65.7 (11-C), 60.7 (7-C), 39.8 (2-C) ppm.

1-Amino-8-(benzyloxycarbonylamino)-3,6-dioxaoctane (4)

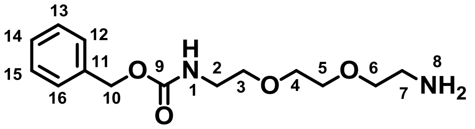

The synthesis was done according to literature under argon.[5] To a solution of 1,8-diamino-3,6-dioxaoctane (11.5 mL, 80.0 mmol) in dry toluene (5 mL) was added a solution of benzyl chloroformiate (1.20 mL, 8.00 mmol) in dry toluene (20 mL) over a period of 8 h at room temperature. After stirring the solution overnight at room temperature, the solvent was evaporated and the residue was dissolved in water (50 mL). The aqueous phase was extracted with DCM (4 × 20 mL), washed with water (3 × 50 mL) and brine (1 × 50 mL) and dried over $MgSO_4$. After filtration and evaporation of the solvent under reduced pressure, the product was obtained.

Molecular formula: $C_{14}H_{22}N_2O_4$ (colorless oil).

Yield: 1.97 g (6.99 mmol, 87%).

R_f value ($CHCl_3$:MeOH:NEt_3 = 9:1:0.2): 0.23.

ESI-HRMS (MeOH) (m/z): Calculated for $[C_{14}H_{22}N_2O_4H]^+$: 283.1652; found: 283.1690.

---

[2] a) Sato, H.; Hayashi, E.; Yamada, N.; Yatagai, M.; Takahara, Y.; *Bioconjugate Chem.* **2001**, *12*, 701; b) Collet, M.; Lenger, J.; Jenssen, K.; Plattner, H. P.; Sewald, N.; *J. Biotechnol.* **2007**, *129*, 316.

^{1}H NMR (300 MHz, $CDCl_3$, 298 K): δ = 7.38 – 7.13 (m, 5H, 12,13,14,15,16-H), 5.52 (s, 1H, 1-NH), 5.01 (d, *J* = 4.0 Hz, 2H, 10-H), 3.62 – 3.37 (m, 10H, 3,4,5,6,7-H), 3.37 – 3.20 (m, 2H, 2-H), 2.77 (br-s, 2H, 8-NH_2) ppm.

^{13}C NMR (75 MHz, $CDCl_3$, 298 K): δ = 156.7 (9-C), 135.6 (11-C), 129.0 (13,15-C), 128.8 (14-C), 128.5 (12,16-C), 70.5 (7-C), 70.0 (3-C), 69.3 (4-C), 69.2 (5-C), 66.4 (10-C), 41.8 (7-C), 40.6 (2-C) ppm.

1,2,3,4,6-Penta-*O*-acetyl-β-D-glucopyranoside (5)

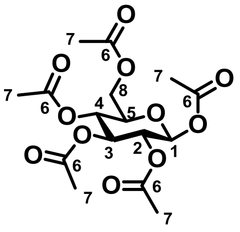

The synthesis was performed as described in literature.[3] A suspension of anhydrous sodium acetate (2.51 g, 30.6 mmol) and acetic anhydride (30.0 mL, 320 mmol) was refluxed for 5 minutes. After addition of D-(+)-glucose (5.02 g, 27.9 mmol) refluxing was continued for further 30 min. The hot solution was poured on ice water (300 mL) and the mixture was extracted with DCM (3 × 50 mL). Drying with $MgSO_4$, filtering, evaporating of the solvent under reduced pressure and drying in high vacuum gave the desired product.

Molecular formula: $C_{16}H_{22}O_{11}$ (brown highly viscous oil).

Yield: 7.50 g (27.9 mmol, 87%).

ESI-HRMS (MeOH) (m/z): Calculated for $[C_{16}H_{22}O_{11}Na]^+$: 413.1054; found: 413.1059.

^{1}H NMR (400 MHz, $CDCl_3$, 298 K): δ = 5.68 (d, *J* = 8.3 Hz,1H, 1-H), 5.40 (dd, 1H, *J* = 3.4, 0.9 Hz, 1H, 2-H), 5.03-5.25 (m, 2H, 2,3-H), 4.1(dd, 2H, *J* = 12.52, 4.49 Hz, 8-H), 3.75-3.81 (m, 1H, 5-H), 2.14-1.07 (m, 15H, 5×7-H) ppm.

^{13}C NMR (101 MHz, $CDCl_3$, 298 K): δ = 170.4-169.0 (5×6-C), 92.2 (1-C), 71.8 (5-C), 70.9 (2-C), 67.0 (3-C), 66.9 (4-C), 61.1 (8-C), 20.9-20.6 (5×7-C) ppm.

2-{2-[2-(Benzyloxycarbonylamino)ethoxy]ethoxy}ethyl-2,3,4,6-penta-*O*-acetyl-β-D-glucopyranoside (6)

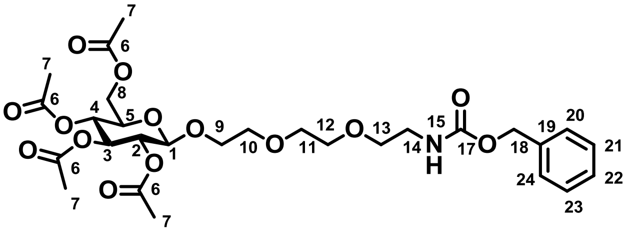

The synthesis was performed as described in literature under an atmosphere of argon.[4] A solution of **5** (551 mg, 1.78 mmol) and **3** (459 mg, 1.62 mmol) in dry DCM (10 mL) was cooled to 0 °C and BF_3*$(OEt)_2$ (0.958 mL, 8.10 mmol) was added drop-wise. After stirring for at 0 °C for 1 h, stirring was continued overnight at room temperature. The reaction

---

[3] Cohen, R. B.; Tsou, K. C.; Rutenburg, S. H.; Seligman, A. M.; *J. Biol. Chem.* **1952**, *195*, 239.
[4] Michel, O.; Ravoo, B. J. *Langmuir* **2008**, *24*, 12116.

mixture was extracted with sat. $NaHCO_3$ (3 × 50 mL) and the aqueous phase was washed with DCM (3 × 50 mL). Drying of the organic phase over $MgSO_4$, filtration and evaporation of the solvent under reduced pressure resulted in a yellow oil, that was purified by silica gel column chromatography using Et_2O:EtOAc = 7:3 as eluent.

Molecular formula: $C_{28}H_{39}NO_{14}$ (colorless oil).

Yield: 324 mg (0.557 mmol, 34%).

R_f value (Et_2O:EtOAc = 7:3): 0.54.

ESI-HRMS (MeOH) (m/z): Calculated for $[C_{28}H_{39}NO_{14}Na]^+$: 636.2263; found: 636.2288.

^{1}H NMR (300 MHz, $CDCl_3$, 298 K): δ = 7.50 – 7.23 (m, 5H, 20,21,22,23,24-H), 5.37 (br-s, 1H, 15-H), 5.31 – 4.94 (m, 5H, 2,3,4,21-H), 4.59 (d, *J* = 7.8 Hz, 1H, 1-H), 4.27 (dd, *J* = 12.3, 4.5 Hz, 1H, 8a-H), 4.15 (dd, *J* = 12.3, 2.1 Hz, 1H, 8b-H), 4.06 – 3.87 (m, 1H, 5-H), 3.84 – 3.51 (m, 10H, 10,11,12,13,14-H), 3.51 – 3.33 (m, 2H, 9-H), 2.11 - 2.02 (m, 12H, 4 × 7-H) ppm.

^{13}C NMR (75 MHz, $CDCl_3$, 298 K): δ = 170.7-169.4 (5×6-C), 156.5 (17-C), 136.6 (19-C), 128.5 (20,21,23,24-C), 128.1 (20-C), 100.8 (1-C), 72.8 (10-C), 71.8 (5-C), 71.2 (2-C), 70.7 (3-C), 70.3 (4-C), 70.1 (8-C), 69.0 (11,12-C), 68.4 (13-C), 66.7 (18-C), 62.0 (9-C), 40.9 (14-C), 20.7-20.6 (4 × 7-C) ppm.

2-[2-(2-Aminoethoxy)ethoxy]ethyl β-D-glucopyranoside (7)

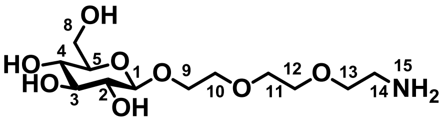

The synthesis was performed as described in literature under an atmosphere of argon.[5] To a solution of **6** (327 mg, 533 μmol) in dry MeOH (10 mL) was added a solution of sodium methoxide (cat. amounts) in dry MeOH (3 mL). After stirring for 2 h at room temperature, DOWEX HCR-W2 (H^+, washed with MeOH) was added until a neural pH was reached. The suspension was filtered and washed with MeOH (10 mL). To this, palladium on carcoal (32 mg, 10 wt %) was added and the suspension was hydrogenated for 24 h at room temperature. After filtration over Celite® to remove the catalyst and washing with MeOH (100 mL), the solvent was evaporated and the residue was dried in high vacuum to give the desired product.

Molecular formula: $C_{12}H_{25}NO_8$ (colorless oil).

Yield: 166 mg (0.533 mmol, quant.).

ESI-HRMS (MeOH) (m/z): Calculated for $[C_{12}H_{25}NO_8H]^+$: 312.1653; found: 312.1654.

^{1}H NMR (300 MHz, MeOD, 298 K): δ = 4.31 (dd, *J* = 7.7, 1.8 Hz, 1H, 1-H), 4.10 – 3.97 (m, 1H, 2-H), 3.88 (dd, *J* = 11.9, 1.6 Hz, 1H, 3-H), 3.83 – 3.58 (m, 3H, 4,8-H), 3.58 – 3.52 (m, 1H, 5-H), 3.50 – 3.28 (m, 8H, 10,11,12,13-H), 3.26 – 3.17 (m, 1H, 4-H), 2.90 – 2.73 (m, 1H, 14-H), 1.10 (d, *J* = 6.3 Hz, 2H, 15-NH_2) ppm.

^{13}C NMR (75 MHz, MeOD, 298 K): δ = 104.5 (1-C), 78.3 (5-C), 78.0 (2-C), 75.1 (3-C), 73.3 (4-C), 71.6 (8-C), 71.5 (11-C), 71.2 (12-C), 70.8 (13-C), 69.6 (10-C), 62.7 (9-C), 42.0 (14-C) ppm.

---

[5] Zemplén, G.; Pacsu, E.; *Ber. Dtsch. Chem. Ges.* **1929**, *62*, 1613.

1,2,3,4,6-Penta-*O*-acetyl β-D-galactopyranoside (8)

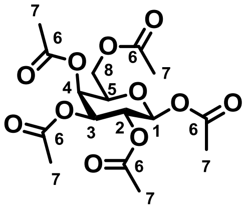

The synthesis was performed as described in literature.[7] A suspension of anhydrous sodium acetate (5.00 g, 60.0 mmol) and acetic anhydride (60.0 mL, 640 mmol) was refluxed for 5 minutes. After addition of D-(+)-galactose (10.0 g, 60.0 mmol) refluxing was continued for further 30 min. The hot solution was poured on ice water (300 mL) and the mixture was extracted with DCM (3 × 50 mL). Drying with $MgSO_4$, filtering, evaporating of the solvent under reduced pressure and drying in high vacuum gave the desired product.

Molecular formula: $C_{16}H_{22}O_{11}$ (brown highly viscous oil).

Yield: 22.8 g (58.4 mmol, 98%).

ESI-HRMS (MeOH) (m/z): Calculated for $[C_{16}H_{22}O_{11}Na]^+$: 413.1054; found: 413.1067.

^{1}H NMR (300 MHz, $CDCl_3$, 298 K): δ = 5.64 (d, *J* = 8.3 Hz, 1H, 1-H), 5.54 – 5.41 (m, 1H, 3-H), 5.39 – 5.13 (m, 2H, 2,4-H), 5.02 (dd, *J* = 10.4, 3.4 Hz, 1H, 5-H), 4.33 – 3.97 (m, 2H, 8-H), 2.22 – 1.89 (m, 15H, 5×7-H) ppm.

^{13}C NMR (75 MHz, $CDCl_3$, 298 K): δ = 170.3-169.0 (5×6-C), 92.1 (1-C), 71.7 (5-C), 70.8 (2-C), 67.8 (3-C), 66.8 (4-C), 61.0 (8-C), 22.2-20.5 (5×7-C) ppm.

2-{2-[2-(Benzyloxycarbonylamino)ethoxy]ethoxy}ethyl 2,3,4,6-penta-*O*-acetyl-β-D-galactopyranoside (9)

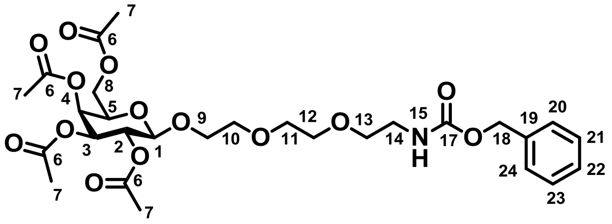

The synthesis was performed as described in literature under an atmosphere of argon.[8] A solution of **8** (1.11 g, 3.60 mmol) and **3** (1.15 g, 4.06 mmol) in dry DCM (20 mL) was cooled to 0 °C and BF_3*$(OEt)_2$ (2.40 mL, 20.3 mmol) was added drop-wise. After stirring for at 0 °C for 1 h, stirring was continued overnight at room temperature. The reaction mixture was extracted with sat. $NaHCO_3$ (3 × 100 mL) and the aqueous phase was washed with DCM (3 × 100 mL). Drying of the organic phase over $MgSO_4$, filtration and evaporation of the solvent under reduced pressure resulted in a yellow oil, that was purified by silica gel column chromatography using Et_2O:EtOAc = 7:3 as eluent.

Molecular formula: $C_{28}H_{39}NO_{14}$ (colorless oil).

Yield: 994 mg (1.62 mmol, 34%).

R_f value (Et_2O:EtOAc = 7:3): 0.58.

ESI-HRMS (MeOH) (m/z): Calculated for $[C_{28}H_{39}NO_{14}Na]^+$: 636.2263; found: 636.2264.

^{1}H NMR (400 MHz, $CDCl_3$, 298 K): δ = 7.40 – 7.07 (m, 5H, 20,21,22,23,24-H), 5.30 (br-s, 1H, 15-H), 5.21 – 5.07 (m, 1H, 2-H), 5.04 (s, 2H, 21-H), 4.98 – 4.88 (m, 1H, 3-H), 4.45 (d,

J = 8.0 Hz, 1H, 1-H), 4.31 – 3.96 (m, 2H, 4,5-H), 3.96 – 3.82 (m, 1H, 8a-H), 3.83 – 3.75 (m, 1H, 8b-H), 3.75 – 3.38 (m, 10H, 10,11,12,13,14-H), 3.38 – 3.22 (m, 2H, 9-H), 2.12 – 1.85 (m, 12H, 4 × 7-H) ppm.

^{13}C NMR (101 MHz, $CDCl_3$, 298 K): δ = 170.4-169.5 (5×6-C), 156.5 (17-C), 136.6 (19-C), 128.5 (20,21,23,24-C), 128.1 (20-C), 101.3 (1-C), 81.4 (10-C), 79.8 (5-C), 76.5 (2-C), 70.9 (3-C), 70.7 (4-C), 70.3 (8-C), 70.1 (11-C), 69.0 (12-C), 68.8 (13-C), 67.0 (18-C), 61.3 (9-C), 40.9 (14-C), 20.8-20.6 (4 × 7-C) ppm.

2-[2-(2-Aminoethoxy)ethoxy]ethyl β-D-galactopyranoside (10)

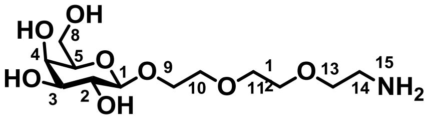

The synthesis was performed as described in literature under an atmosphere of argon.[9] To a solution of **9** (191 mg, 312 μmol) in dry MeOH (5 mL) was added a solution of sodium methoxide (cat. amounts) in dry MeOH (1 mL). After stirring for 2 h at room temperature, DOWEX HCR-W2 (H^+, washed with MeOH) was added until a neural pH was reached. The suspension was filtered and washed with MeOH (10 mL). To this, palladium on carcoal (32 mg, 10 wt %) was added and the suspension was hydrogenated for 24 h at room temperature. After filtration over Celite® to remove the catalyst and washing with MeOH (100 mL), the solvent was evaporated and the residue was dried in high vacuum to give the desired product.

Molecular formula: $C_{12}H_{25}NO_8$ (colorless oil).

Yield: 97.0 mg (0.312 mmol, quant.).

ESI-HRMS (MeOH) (m/z): Calculated for $[C_{12}H_{25}NO_8H]^+$: 312.1653; found: 312.1662.

^{1}H NMR (300 MHz, MeOD, 298 K): δ = 4.17 (d, J = 7.2 Hz, 1H, 1-H), 3.99 – 3.81 (m, 2H, 2,3-H), 3.74 (d, J = 2.3 Hz, 1H, 4-H), 3.71 – 3.31 (m, 13H, 5,8,9,10,11,12,13-H), 2.70 (d, J = 4.9 Hz, 2H, 14-H) ppm.

^{13}C NMR (75 MHz, MeOD, 298 K): δ = 105.0 (1-C), 76.7 (5-C), 74.9 (2-C), 72.5 (3-C), 71.4 (4-C), 71.3 (8-C), 71.1 (11,12-C), 70.4 (13-C), 69.6 (10-C), 62.5 (9-C), 42.0 (14-C) ppm.

1,2,3,4,6-Penta-*O*-acetyl β-D-mannopyranoside (11)

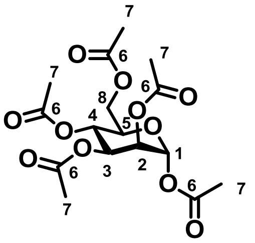

The synthesis was performed as described in literature.[7] A suspension of anhydrous sodium acetate (5.00 g, 60.0 mmol) and acetic anhydride (60.0 mL, 640 mmol) was refluxed for 5 minutes. After addition of D-(+)-mannose (10.0 g, 60.0 mmol) refluxing was continued for further 30 min. The hot solution was poured on ice water (300 mL) and the mixture was extracted with DCM (3 × 50 mL). Drying with $MgSO_4$, filtering, removing the solvent under reduced pressure gave the crude product which was dissolved in DCM (100 mL) and washed

with sat. $NaHCO_3$ (3 × 50 mL). After drying over $MgSO_4$, filtration, evaporation of the solvent under reduced pressure and drying in high vacuum, the product was obtained.

Molecular formula: $C_{16}H_{22}O_{11}$ (brown highly viscous oil).

Yield: 23.2 g (59.4 mmol, 99 %).

ESI-HRMS (MeOH) (m/z): Calculated for $[C_{16}H_{22}O_{11}Na]^+$: 413.1054; found: 413.1036.

^{1}H NMR (300 MHz, $CDCl_3$, 298 K): δ = 6.02 (d, *J* = 1.8 Hz, 1H, 1-H), 5.42 (dd, *J* = 3.2, 1.1 Hz, 1H, 2-H), 5.36 – 5.15 (m, 2H, 3,4-H), 5.08 (dd, *J* = 10.0, 3.3 Hz, 1H, 5-H), 4.13 – 3.91 (m, 2H, 8-H), 2.35 – 1.82 (m, 15H, 5×7-H) ppm.

^{13}C NMR (75 MHz, $CDCl_3$, 298 K): δ = 170.3-169.0 (5×6-C), 92.1 (1-C), 71.7 (5-C), 70.8 (2-C), 67.8 (3-C), 66.8 (4-C), 61.0 (8-C), 22.2-20.5 (5×7-C) ppm.

2,3,4,6-Tetra-*O*-acetyl-α/β-D-mannopyranosyl trichloroacetimidate (12)

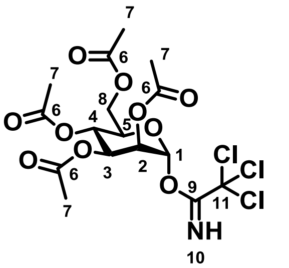

The synthesis was performed as described in literature under an atmosphere of argon.[6] A solution of **11** (3.00 g, 7.68 mmol) and hydrazine acetate (850 mg, 9.22 mmol) in dry DMF (15 mL) was stirred at 60 °C for 1 h. The mixture was diluted with EtOAc (50 mL), washed with water (3 × 30 mL) and brine (1 × 30 mL), dried over $MgSO_4$ and evaporated to dryness under reduced pressure to yield a yellow oil. After dissolving the oil in dry DCM (10 mL), trichloroacetimidate (7.70 mL, 76.8 mmol) were added and the solution was cooled to 0 °C. The solution was treated with 1,8-diazabicycloundec-7-ene (DBU) (0.152 mL, 768 μmol) and stirred at 0 °C for 1 h. After warming to room temperature, stirring was continued for another hour and then the solvents were removed under reduced pressure. The brown oil was purified by silica gel column chromatography using cyclohexane:EtOAc = 1:1 as eluent.

Molecular formula: $C_{16}H_{20}Cl_3NO_{10}$ (highly viscous yellowish oil).

Yield: 1.69 g (3.43 mmol, 45%).

R_f value (cyclohexane:EtOAc = 1:1): 0.67.

ESI-HRMS (MeOH) (m/z): Calculated for $[C_{16}H_{20}Cl_3NO_{10}Na]^+$: 514.0045; found: 514.0045.

^{1}H NMR (400 MHz, $CDCl_3$, 298 K): δ = 8.79 (s, 1H, 10-H), 6.27 (d, *J* = 1.8 Hz, 1H, 1-H), 5.68 – 5.25 (m, 3H, 2,3,5-H), 4.47 – 3.87 (m, 3H, 4,10-H), 2.14 – 1.87 (m, 12H, 4×7-H) ppm.

^{13}C NMR (101 MHz, $CDCl_3$, 298 K): δ = 170.6-169.6 (4×6-C), 159.7 (9-C), 94.5 (1-C), 90.5 (11-C), 71.2 (5-C), 68.8 (2-C), 67.8 (3-C), 65.3 (4-C), 62.0 (8-C), 21.0- 20.6 (4×8-C) ppm.

[6] Cheng, H.; Cao, X.; Xian, M.; Fang, L.; Cai, T. B.; Ji, J. J.; Tunac, J. B.; Sun, D.; Wang, P. G.; *J. Med. Chem.* **2005**, *48*, 645.

2-{2-[2-(Benzyloxycarbonylamino)ethoxy]ethoxy}ethyl 2,3,4,6-tetra-*O*-acetyl-α-D-mannopyranoside (13)

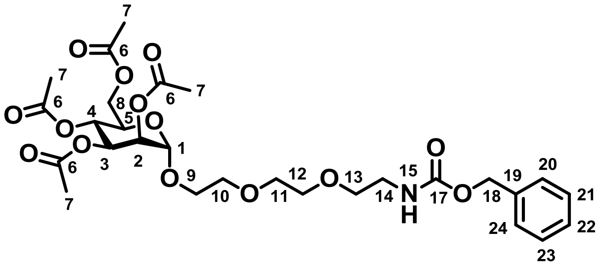

The synthesis was performed as described in literature under an atmosphere of argon.[7] Powdered mol sieve (4 Å, 80.0 mg) was added to a solution of **12** (500 mg, 1.01 mmol) and **3** (431 mg, 1.52 mmol) in dry DCM (10 mL). After cooling the mixture to −25 °C, trimethylsilyl trifluoromethanesulfonate (TMSOTf) (0.180 mL, 1.01 mmol) was added drop-wise. Stirring was continued 1 h at −25 °C and then the mixture was warmed to room temperature and stirred overnight. After quenching the reaction by addition of sat. $NaHCO_3$ (20 mL), it was extracted with DCM (3 × 50 mL) and washed with sat. $NaHCO_3$ (3 × 20 mL), water (3 × 20 mL) and brine (1 × 20 mL). Drying over $MgSO_4$, filtering and evaporating the solvent gave the crude product, which was purified by silica column chromatography using cyclohexane:EtOAc = 1:1 as eluent.

Molecular formula: $C_{28}H_{39}NO_{14}$ (colorless oil).

Yield: 192 mg (0.313 mmol, 31%).

R_f value (cyclohexane:EtOAc = 1:1): 0.14.

ESI-HRMS (MeOH) (m/z): Calculated for $[C_{28}H_{39}NO_{14}Na]^+$: 636.2263; found: 636.2258.

^{1}H NMR (400 MHz, $CDCl_3$, 298 K): δ = 7.71 – 7.22 (m, 5H, 20,21,22,23,24-H), 5.29 (dd, *J* = 10.0, 3.4 Hz, 1H, 2-H), 5.21 – 5.17 (m, 1H, 1-H), 5.03 (s, 2H, 18-H), 4.80 (s, 1H, 3-H), 4.25 – 4.11 (m, 1H, 8a-H), 4.04 – 3.93 (m, 2H, 4,8b-H), 3.78 – 3.66 (m, 1H, 5-H), 3.66 – 3.35 (m, 11H, 10,11,12,13,14-H), 3.37 – 3.19 (m, 2H, 9-H), 2.10 – 1.80 (m, 12H, 4×7-H) ppm.

^{13}C NMR (101 MHz, $CDCl_3$, 298 K): δ = 170.7-169.7 (5×6-C), 156.5 (17-C), 136.7 (19-C), 128.6 (21,23-C), 128.5 (20,22,24-C), 97.7 (1-C), 70.6 (13-C), 70.3 (5-C), 70.1 (2-C), 70.0 (10-C), 69.6 (11-C), 69.01 (12-C), 68.4 (3-C), 66.6 (4-C), 66.1 (18-C), 62.4 (9-C), 40.9 (14-C), 29.7-20.7 (4×7-C) ppm.

2-[2-(2-Aminoethoxy)ethoxy]ethyl α-D-mannopyranoside (14)

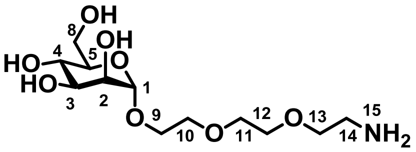

The synthesis was performed as described in literature under an atmosphere of argon.[9] To a solution of **13** (161 mg, 262 μmol) in dry MeOH (4 mL) was added a solution of sodium methoxide (cat. amounts) in dry MeOH (1 mL). After stirring for 2 h at room temperature, DOWEX HCR-W2 (H^+, washed with MeOH) was added until a neural pH was reached. The suspension was filtered and washed with MeOH (10 mL). To this, palladium on carcoal

---

[7] a) Houseman, B. T.; Mrksich, M.; *Chem. Biol.* **2002**, *9*, 443; b) Chatterjee, M. N.; Kay, E. R.; Leigh, D. A.; *J. Am. Chem. Soc.* **2006**, *128*, 4058.

(16 mg, 10 wt %) was added and the suspension was hydrogenated for 24 h at room temperature. After filtration over Celite® to remove the catalyst and washing with MeOH (100 mL), the solvent was evaporated and the residue was dried in high vacuum to give the desired product.

Molecular formula: $C_{12}H_{25}NO_8$ (colorless oil).

Yield: 81.6 mg (0.262 mmol, quant.).

ESI-HRMS (MeOH) (m/z): Calculated for $[C_{12}H_{25}NO_8H]^+$: 312.1653; found: 312.1658.

^{1}H NMR (300 MHz, MeOD, 298 K): δ = 4.70 (d, *J* = 1.5 Hz, 1H, 1-H), 3.84 – 3.67 (m, 3H, 2,3,4-H), 3.67 – 3.36 (m, 13H, 8,9,10,11,12,13-H), 2.77 – 2.61 (m, 2H, 14-H) ppm.

^{13}C NMR (75 MHz, MeOD, 298 K): δ = 101.8 (1-C), 74.7 (5-C), 73.6 (3-C), 72.5 (2-C), 72.1 (13-C), 71.6 (4-C), 71.4 (10-C), 68.6 (11,12-C), 67.8 (9-C), 62.9 (8-C), 42.1 (14-C) ppm.

1-Amino-8-(benzyloxycarbonylamino)-3,6-dioxaoctane-D-neuraminic acid (15)

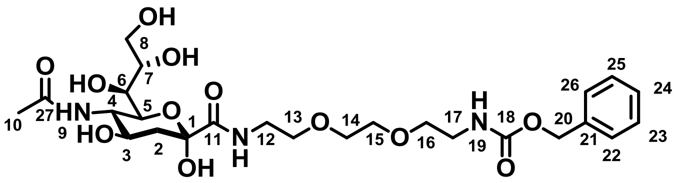

The peptide coupling was performed under an atmosphere of argon. To a solution of D-neuraminic acid (200 mg, 647 μmol) in dry DMF (5 mL) was added Oxyma pure® (110 mg, 776 μmol) and the mixture was stirred for 30 min at room temperature. After addition of **4** (183 mg, 647 μmol) and DIPCDI (0.120 mL, 776 μmol) the orange mixture was stirred for 48 h at room temperature. The solvent was removed under reduced pressure and the residue was purified by silica gel column chromatography using DCM:MeOH = 9:1 → DCM: MeOH = 5:1 as eluent.

Molecular formula: $C_{25}H_{39}N_3O_{12}$ (colorless oil).

Yield: 170 mg (296 μmol, 46%).

R_f value (DCM: MeOH = 5:1): 0.24.

ESI-HRMS (MeOH) (m/z): Calculated for $[C_{25}H_{39}N_3O_{12}Na]^+$: 596.2426; found: 596.2426.

^{1}H NMR (300 MHz, MeOD, 298 K): δ = 7.41 – 7.02 (m, 5H, 22,23,24,25,26-H), 4.97 (s, 2H, 20-H), 4.48 (s, 1H, 3-H,), 4.12 – 3.83 (m, 3H, 4,8-H), 3.84 – 3.66 (m, 4H, 13,16-H), 3.66 – 3.35 (m, 9H, 5,6,7,12,14,15-H), 3.40 – 3.30 (t, *J* = 5.6 Hz, 2H, 17-H), 2.22 – 2.12 (m, 1H, 2a-H), 1.92 (s, 3H, 10-H), 1.48 (dd, *J* = 12.7, 11.3 Hz, 1H, 2b-H) ppm.

^{13}C NMR (75 MHz, MeOD, 298 K): δ = δ 175.0 (27-C), 174.0 (11-C), 159.0 (18-C), 138.4 (21-C), 129.5 (23,25-C), 129.0 (24-C), 128.9 (22,26-C), 96.7 (1-C), 72.3 (7-C), 72.1 (13-C), 71.8 (16-C), 71.3 (6-C), 71.0 (5-C), 70.3 (14-C), 70.2 (15-C), 67.8 (20-C), 67.5 (3-C), 64.8 (8-C), 53.3 (4-C), 42.1 (2-C), 41.7 (17-C), 40.3 (12-C), 22.8 (10-C) ppm.

1,8-Diamino-3,6-dioxaoctane-D-neuraminic acid (16)

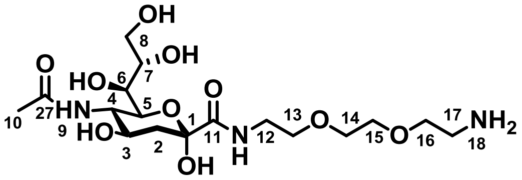

The synthesis was performed under an atmosphere of hydrogen. **15** (170 mg, 296 μmol) was dissolved in dry MeOH (5 mL). After the addition of palladium on carcoal (17 mg, 10 wt %), the suspension was hydrogenated for 24 h at room temperature. After filtration over Celite® to remove the catalyst and washing with MeOH (100 mL), the solvent was evaporated and the residue was dried in high vacuum to give the desired product.

Molecular formula: $C_{17}H_{33}N_3O_{10}$ (colorless oil).

Yield: 81.6 mg (0.262 mmol, quant.).

ESI-HRMS (MeOH) (m/z): Calculated for $[C_{17}H_{33}N_3O_{10}H]^+$: 440.2239; found: 440.2235.

^{1}H NMR (400 MHz, MeOD, 298 K): δ = 4.03 – 3.90 (m, 1H, 3-H), 3.73 (ddd, *J* = 25.3, 14.6, 9.0 Hz, 2H, 4,6-H), 3.64 – 3.38 (m, 13H, 7,8,12,13,14,15,16-H), 3.38 – 3.29 (m, 2H, 5,17a-H), 2.83 – 2.69 (m, 2H, 17b-H), 2.17 (dd, *J* = 13.7, 3.6 Hz, 1H, 2-H_a), 1.93 (s, 3H, 10-H), 1.60 – 1.37 (m, 1H, 2-H_b) ppm.

^{13}C NMR (101 MHz, MeOD, 298 K): δ = 175.0 (27-C), 174.2 (11-C), 97.2 (1-C), 72.5 (7-C), 72.2 (13-C), 71.7 (16-C), 71.3 (5,6-C), 70.2 (14,15-C), 67.9 (3-C), 64.9 (8-C), 54.2 (4-C), 42.2 (2-C), 41.8 (17-C), 40.3 (12-C), 22.9 (10-C) ppm.

N-*tert*-Butoxycarbonyl ethylendiamine (17)

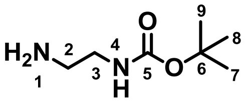

The synthesis was performed as described in literature under an atmosphere of argon.[8] Ethylendiamine (12.2 mL, 183 mmol) was dissolved in dry DCM (160 mL) and the solution was cooled to 0 °C. After drop-wise addition of a solution of di-*tert*-butyl dicarbonate (Boc_2O) (4.00 g, 18.3 mmol) in dry DCM (100 mL) over 3 h at 0 °C, the solution was stirred overnight at room temperature. The reaction mixture was washed with water (4 × 100 mL), dried over $MgSO_4$, filtered and evaporated under reduced pressure. After drying in high vacuum the product was obtained.

Molecular formula: $C_7H_{16}N_2O_2$ (colorless oil).

Yield: 1.71 g (10.7 mmol, 58%).

ESI-HRMS (MeOH) (m/z): Calculated for $[C_7H_{16}N_2O_2H]^+$: 116.1285; found: 116.1287.

^{1}H NMR (300 MHz, $CDCl_3$, 298 K): δ = 4.94 (br-s, 1H, 4-NH), 3.10 (dd, *J* = 11.6, 5.8 Hz, 2H, 3-H), 2.73 (t, *J* = 5.9 Hz, 2H, 2-H), 1.50 – 1.26 (m, 11H, 1-NH_2,7,8,9-H) ppm.

^{13}C NMR (75 MHz, $CDCl_3$, 298 K): δ = 155.2 (5-C), 78.2 (6-C), 42.4 (3-C), 40.9 (2-C), 27.4 (7,8,9-C) ppm.

[8] Onajole, O. K; Govender, P.; Govender, T.; Maguire, G. E. M.; Kruger, H. G.; *Struct Chem.* **2009**, *20*, 1067.

1-[*N*-(9-Fluorenylmethoxycarbonyloxy)]-4-[*N*-*tert*-butoxycarbonyl]ethylenediamine (18)

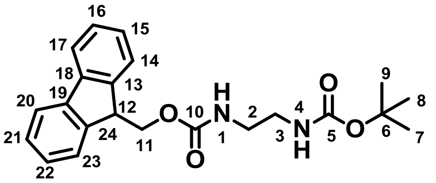

The synthesis was performed as described in literature.[9] To a solution of **17** (1.68 g, 10.5 mmol) and $NaHCO_3$ (1.35 g, 16.1 mmol) in a mixture of water (60 mL) and ACN (60 mL) was added drop-wise a solution of *N*-(9-fluorenylmethoxycarbonyloxy)succinimide (Fmoc-OSu) (3.19 g, 9.45 mmol) in ACN (50 mL). The suspension was stirred for 4 h at room temperature and DCM (200 mL) was added. After separation of the phases, the aqueous phase was extracted with DCM (2 × 100 mL) and the organic phases were washed with water (3 × 100 mL) and brine (1 × 50 mL). The organic phase was dried over $MgSO_4$, filtered, evaporated under reduced pressure and dried in high vacuum to give the product.

Molecular formula: $C_{22}H_{26}N_2O_4$ (white solid).

Yield: 3.60 g (9.40 mmol, 90%).

ESI-HRMS (MeOH) (m/z): Calculated for $[C_{22}H_{26}N_2O_4Na]^+$: 405.1785; found: 405.1785.

^{1}H NMR (300 MHz, $CDCl_3$, 298 K): δ = 7.68 (d, *J* = 7.5 Hz, 2H, 17,20-H), 7.52 (t, *J* = 8.9 Hz, 2H, 14,24-H), 7.42 – 7.14 (m, 4H, 15,16,21,22-H), 5.22 (d, *J* = 5.8 Hz, 1H, 1-NH), 4.82 (s, 1H, 4-NH), 4.37 (dd, *J* = 36.9, 14.6 Hz, 2H, 11-H), 4.12 (t, *J* = 6.7 Hz, 1H, 12-H), 3.48 – 2.87 (m, 4H, 2,3-H), 1.36 (s, 9H, 7,8,9-H) ppm.

^{13}C NMR (75 MHz, $CDCl_3$, 298 K): δ = 155.8 (10-C), 155.5 (5-C), 142.9 (13, 24-C), 140.3 (18,19-C), 126.7 (15,22-C), 126.0 (16,21-C), 124.0 (14,23-C), 118.9 (17,20-C), 78.6 (6-C), 65.7 (11-C), 46.2 (12-H), 40.6 (3-C), 39.5 (2-C), 27.4 (7,8,9-C) ppm.

***N*-(9-Fluorenylmethoxycarbonyloxy)diaminoethane hydrochloride (19)**

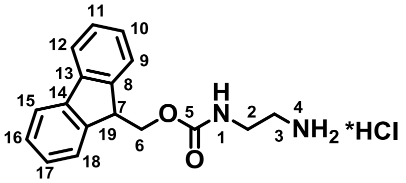

The synthesis was performed as described in literature.[15] **18** (3.50 g, 9.15 mmol) was dissolved in EtOAc p.a. (200 mL) and 18 M HCl (5.10 mL, 91.5 mmol) was added dropwise. After stirring for 4 h at room temperature, the formed precipitate was collected by filtration, washed with EtOAc (300 mL) and dried in high vacuum to give the product.

Molecular formula: $C_{17}H_{19}N_2O_2$*HCl (white solid).

Yield: 2.68 g (8.38 mmol, 92%).

ESI-HRMS (MeOH) (m/z): Calculated for $[C_{17}H_{19}N_2O_2Na]^+$: 283.1447; found: 283.1442.

^{1}H NMR (300 MHz, DMSO-d_6, 298 K): δ = 8.20 (s, 3H, 4-NH_3^+), 7.87 (d, *J* = 7.3 Hz, 2H, 12,15-H), 7.69 (d, *J* = 7.3 Hz, 2H, 9,18-H), 7.54 (t, *J* = 5.6 Hz, 1H, 1-NH), 7.49 – 7.24 (m, 4H,

[9] Mandal, P. K.; Ren, Z.; Chen, X.; Xiong, C.; McMurray, J. S.; *J. Med. Chem.* **2009**, *52*, 6126

10,11,16,17-H), 4.36 – 4.14 (m, 3H, 6,7-H), 3.26 (q, *J* = 6.3 Hz, 2H, 2-H), 2.83 (dd, *J* = 11.3, 5.5 Hz, 2H, 3-H) ppm.

^{13}C NMR (75 MHz, DMSO-d$_6$, 298 K): δ = 161.5 (5-C), 149.0 (8,19-C), 146.0 (13,14-C), 132.8 (10,17-C), 132.3 (11,16-C), 130.4 (9,18-C), 125.3 (12,15-C), 70.8 (6-C), 51.9 (7-C), 43.7 (2-C), 43.2 (3-C) ppm.

1-(9*H*-Fluoren-9-yl)-3,8-dioxo-2,10,13,16-tetraoxa-4,7-diazaoctadecan-18-oic acid (20)

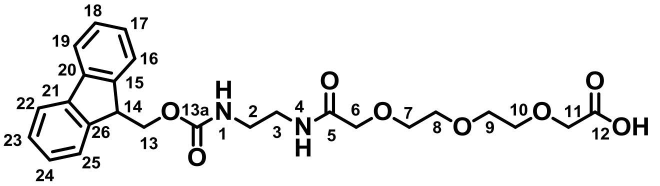

The peptide coupling was performed under an atmosphere of argon. To a solution of 3,6,9-trioxaundecanedioic acid (7.02 mL, 41.1 mmol) in dry DCM (40 mL) was added a solution of Oxyma pure® (584 mg, 4.11 mmol) and DIPCDI (0.640 mL, 4.11 mmol) in dry DCM (20 mL) drop-wise. To the orange reaction mixture was added a suspension of **19** (1.31 g, 4.11 mmol) and *N*-methylmorpholine (NMM) (0.450 mL, 4.11 mmol) in dry DCM (40 mL) drop-wise. After the addition was finished, the suspension was stirred overnight at room temperature. The reaction mixture was washed with 5 % citric acid (4 × 100 mL), dried over $MgSO_4$, filtered and evaporated under reduced pressure. After drying in high vacuum, the product was obtained.

Molecular formula: $C_{25}H_{30}N_2O_8$ (colorless oil).

Yield: 1.80 g (3.70 mmol, 90%).

ESI-HRMS (MeOH) (m/z): Calculated for $[C_{25}H_{29}N_2O_8]^-$: 485.1929; found: 485.1929.

^{1}H NMR (300 MHz, CDCl$_3$, 298 K): δ = 7.68 (d, *J* = 7.5 Hz, 2H, 19,22-H), 7.52 (d, *J* = 7.4 Hz, 2H, 16,24-H), 7.36 – 7.15 (m, 5H, 17,18,24,25-H, 4-NH), 5.87 (t, *J* = 5.6 Hz, 1H, 1-NH), 5.23 (s, 2H, 13-H), 4.33 4.44 – 4.22 (m, 4H, 6,11-H), 4.24 – 3.99 (m, 1H, 23-H), 3.84 – 3.49 (m, 8H, 7,8,9,10-H), 3.33 (dq, *J* = 17.3, 6.0 Hz, 4H, 2,3-H) ppm.

^{13}C NMR (75 MHz, CDCl$_3$, 298 K): δ = 172.4 (12-C), 171.6 (5-C), 158.7 (13a-C), 143.8 (15,28-C), 141.3 (20,21-C), 127.7 (17,24-C), 127.1 (18,23-C), 125.1 (16,15-C), 71.1 (8,9-C), 70.7 (7,10-C), 70.1 (6-C), 70.0 (11-C), 67.0 (13-C), 47.1 (14-C), 40.4 (2-C), 38.8 (3-C) ppm.

Solid phase peptide synthesis (SPPS)

Standard operation procedure loading of the resin (SOP 1): Loading of the resin was performed according to literature under an atmosphere of argon.[10] The according amino acid (2 equiv relative to resin loading) was dissolved in dry DCM and a small amount of DMF (SPPS grade) and is added to 2-chlorotrityl resin (loading: 1.50 mmol/g). After addition of DIPEA (2 equiv relative to resin loading) the mixture is agitated by a slow stream of argon for 5 min. Following another addition of DIPEA (3 equiv relative to resin loading), agitation is continued for 1 h. The remaining reactive groups of the resin are quenched with MeOH p.a. (1 mL/g resin) for 15 min. After filtration of the resin it was washed consecutively with DCM

[10] A) Barlos, K.; Gatos, D.; Kallitsis, J.; Papaphotiu, G.; Sotiriu, P.; Wenqing, Y.; Schafer, W.; *Tetrahedron Lett.* **1989**, *30*, 3943; b) Barlos, K.; Chatzi, O.; Gatos, D.; Stavropoulos, G.; *Int. J. Peptide Protein Res.* **1991**, *37*, 513; c) Bollhagen, R.; Schmiedberger, M.; Barlos, K.; Grell, E.; *Chem. Commun.* **1994**, 2559.

p.a. (3 × 20 mL), DMF SPPS grade (3 × 20 mL), DCM p.a. (3 × 20 mL) and MeOH p.a. (3 × 20 mL) and dried in high vaccum until a constant weight is reached.

Determination of the beads´ loading[11] was done by suspending 6.00 mg dry beads in 20% piperidine in DMF (1 mL) and stirring for 20 min at room temperature. 100 µL of the supernatant were diluted with DMF p.a. (10 mL) and the absorbance from 290 to 310 nm is measured against DMF p.a. Loading can be calculated by using the following formulas:

$$loading\ \left(\frac{mmol}{g}\right) = I(295\,nm) * \frac{142}{mg}$$

$$loading\ \left(\frac{mmol}{g}\right) = I\ (304\,nm) * \frac{164}{mg}$$

Standard operation procedure for step-wise chain elongation via SPPS (SOP 2): SPPS was performed according to literature.[1,2] Dry beads were swelled in DMF p.a. for 45 min while shaking the reaction vessel. After sucking of the DMF p.a., the Fmoc-group is cleaved with 20% piperidine in DMF for 20 min. The resin is washed with DMF p.a. (4 times) and alternately with DCM p.a. (3 times) and isopropanole p.a. (3 times) to fully remove the piperidine. Success of the cleavage was controlled by the Kaiser test. Equal amounts of a 5% ninhydrin solution in EtOH, 80% phenol in EtOH and 0.001 M KCN in pyridine were mixed with a few washed beads and heated for 1 min to 100 °C. A blue color of the beads indicated free amine functions on the resin. The coupling step was performed by suspending the resin in a solution of the according Fmoc-protected amino acid (3 equiv) and Oxyma pure® (3.6 equiv) in DMF (SPPS grade). After addition of DIPCDI (3.3 equiv), the suspension was shaken for 2 h and then the solvent was sucked off. Washing of the beads with DMF (SPPS grade, 4 times) was followed by the Kaiser test which should not result in a color change if the coupling was successful. The deprotection and coupling steps were continued until the desired peptide sequence was obtained.

Standard operation procedure for cleavage of resin and removal of permanent protection groups (SOP 3): Cleavage of the resin and removal of permanent protection groups was performed according to literature. [1,12] Prior to splitting of the peptide from the resin, the Fmoc-group was removed by treatment with 20% piperidine in DMF for 20 min. After washing with DMF p.a. (4 times) and alternately with DCM (2 times) and isopropanole p.a. (2 times), the beads were suspended in a solution of TFA:H_2O:EDT:TIS = 94:2.5:2.5:1 and stirred for 5 h at rt. After filtration, the beads were washed with TFA (5 times) and the filtrate was concentrated until the peptide started to precipitate. Addition of cold Et_2O resulted in complete precipitation and the suspension was keep overnight in the freezer. The peptide was collected by filtration, washed with Et_2O and dried in high vacuum to yield the peptides as white, hygroscopic solids which were stored under argon.

[11] Blanco-Canosa, J.B.; Dawson, P.E.; *Angew. Chem. Int. Ed.* **2008**, *47*, 6851.
[12] Carpino, L.A.; El-Faham, A.; *Tetrahedron* **1999**, *55*, 6813.

FITC-Cys-His-Cys (21)

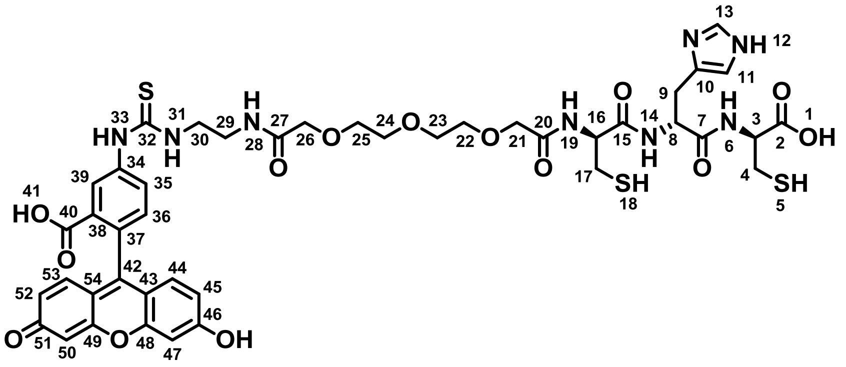

The synthesis was performed as described in SOP 1, SOP 2 and SOP 3 using Fmoc-Cys(Trt)-OH, Fmoc-His(Trt)-OH, Fmoc-Cys(Trt)-OH, **19** and fluorescein isothiocyanate. In case of **19** coupling was done overnight. Incorporation of the fluorescein moiety was achieved according to literature[13] by using fluorescein isothiocyanate (1.5 equiv) and DIPEA (3.0 equiv) in DMF SPPS grade overnight.

Molecular formula: $C_{43}H_{48}N_8O_{13}S_3$ (yellow solid).

Yield (overall): 892 mg (909 μmol, 61%).

ESI-HRMS (MeOH) (m/z): Calculated for $[C_{43}H_{48}N_8O_{13}S_3H]^+$: 1019.2345; found: 1019.2355.

^{1}H NMR (400 MHz, DMSO-d$_6$, 298 K): δ = 14.22 (br-s, 1H, 41-H), 10.21 (br-s, 1H, 1-H), 8.96 (d, *J* = 1.3 Hz, 1H, 11-H), 8.52 (d, *J* = 8.0 Hz, 1H, 6-NH), 8.43 – 8.29 (m, 1H, 12-NH), 8.28 – 8.12 (m, 2H, 33-NH,39-H), 8.02 – 7.88 (m, 1H, 28-H), 7.83 (d, *J* = 7.7 Hz, 1H, 35-H), 7.73 (br-d, *J* = 6.9 Hz, 1H, 14-H), 7.41 – 7.21 (m, 3H,13-H, 19,31-NH), 7.18 (d, *J* = 8.3 Hz, 1H, 36-H), 6.68 (d, *J* = 2.3 Hz, 2H, 44,53-H), 6.65 – 6.49 (m, 4H, 45,47,50,52-H), 4.66 (dd, *J* = 13.7, 8.2 Hz, 1H, 8-H), 4.47 – 4.38 (m, 2H, 3,16-H), 4.01 – 3.87 (m, 4H, 21,26-H), 3.69 – 3.47 (m, 8H, 22,23,24,25-H), 3.22 – 2.70 (m, 6H, 4,9,17-H), 2.43 (t, *J* = 8.6 Hz, 1H, 5-SH), 2.29 (t, *J* = 8.5 Hz, 1H, 18-SH) ppm.

^{13}C NMR (101 MHz, DMSO-d$_6$, 298 K): δ = 180.7-168.5 (2,7,15,20,27,32,40,51-C), 159.5 (46-C), 151.9 (48,49-C), 144.1 (34,42-C), 141.1 (11-C), 133.6 (10,37,38-C), 129.0 (36-C), 126.6 (53-C), 124.1 (44-C), 117.1-116.9 (13,35,39,43,45-C), 112.5 (54-C), 109.8 (52-C), 102.2 (47,50-C), 70.2 (23,24-C), 69.9 (22-C), 69.7 (25-C), 69.5 (21,26-C), 54.4 (16-C), 54.3 (8-C), 51.6 (3-C), 43.6 (30-C), 37.5 (29-C), 26.6 (9-C), 26.0 (18-C), 25.4 (4-C) ppm.

[13] Song, A.; Wang, X.; Zhang, J.; Marik, J.; Lebrillab, C. B.; Lama, K. S.; *Bioorg. Med. Chem. Lett.* **2004**, *14*, 161.

FITC-HisHis (22)

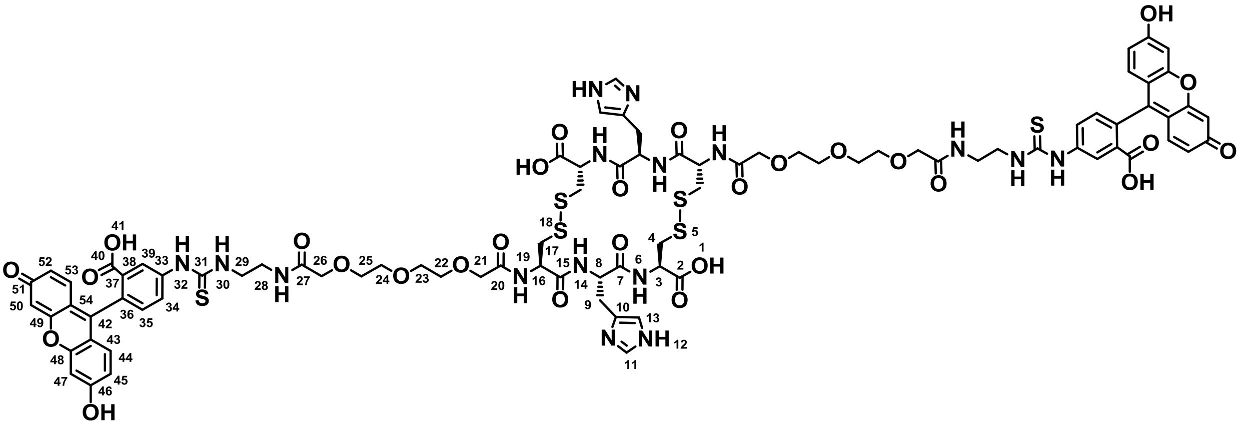

The appropriate amount of labeled tripeptide **21** was dissolved in phosphate buffer (100 mM, pH 7.4) and stirred in an open vial until the *Ellman*-test was negative. The resulting dimer was used without further purification for ITC and fluorescence measurements.

Molecular formula: $C_{86}H_{92}N_{16}O_{28}S_6$(white solid).

ESI-HRMS (MeOH) (m/z): Calculated for $[C_{86}H_{92}N_{16}O_{28}S_6H_2]^{2+}$: 995.2369; found: 995.2375.

Isothermal titration calorimetry of FITC-HisHis with NANA

ITC measurements were performed using a NanoITC system (Calorimetry Sciences Cooperation, USA) and ITC Run software. Sample solutions were prepared by dissolving the appropriate amount of FITC-HisHis and NANA in phosphate buffer (100 mM, pH 7.4). Before filling the cell, the solution was degassed for 30 min. A solution of 2 mM FITC-HisHis was titrated with a degassed solution (30 min) of 40 mM NANA. All measurements were performed at 23 °C using a stirring rate of 300 rpm and a 400 s interval between each injection. To determine the heat of dilution, a NANA solution was titrated into phosphate buffer (100 mM, pH 7.4). The heat of dilution was subtracted from the raw heat data. The data were fitted to a 1:2 model using a spread sheet method.[14]

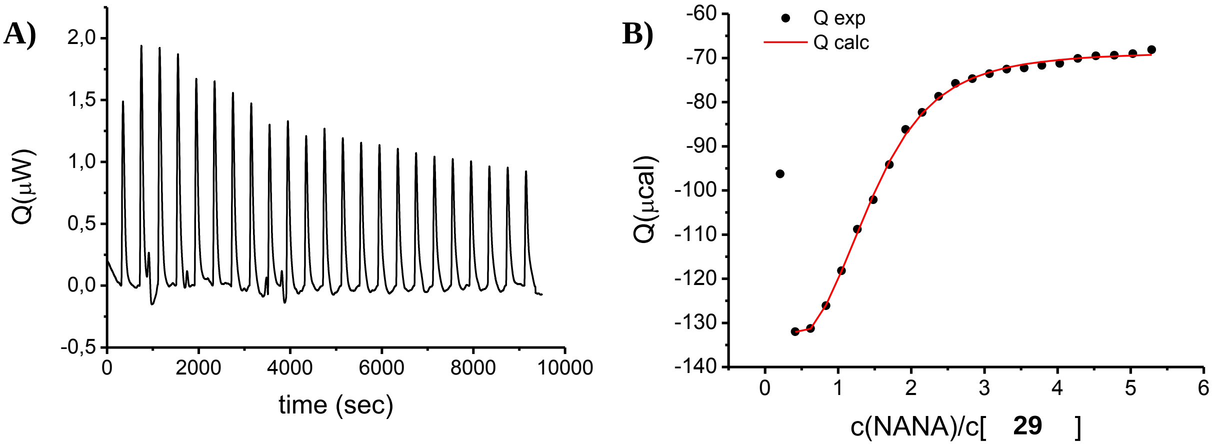

Figure S1: A) Raw data of ITC titration of 2 mM FITC-HisHis with 40 mM NANA in phosphate buffer (100 mM, pH 7.4); B) Fit obtained from the raw data shown in A) (first data point is not used for the fit).

TableS1: ITC data for the interaction of HisHis and FITC-HisHis with NANA.

	n[a]	K [M^{-1}]	ΔG [kJ mol^{-1}]	ΔH [kJ mol^{-1}]	ΔS [J mol^{-1} K^{-1}]
HisHis	2	K_1=72.7	ΔG_1= −10.6	ΔH_1= −6.27	ΔS_1= 14.6
		K_2=7.76×10^3	ΔG_2= −22.2	ΔH_2= −1.54	ΔS_2= 69.4
FITC-HisHis	2	K_1=163	ΔG_1= −12.6	ΔH_1= −2.70	ΔS_1= 9.93
		K_2=5.36×10^3	ΔG_2= −21.3	ΔH_2= −2.79	ΔS_2= 62.1

(Data for HisHis are taken from M. Rauschenberg et al. *Angew. Chem. Int Ed.* **2010**, *49*, 7340)

14 Huskens J., van Bekkum H., Peters J. A., *Computers & Chemistry*, **1995**, *19*, 409 – 416.

UV–vis spectrum of FITC-Cys-His-Cys

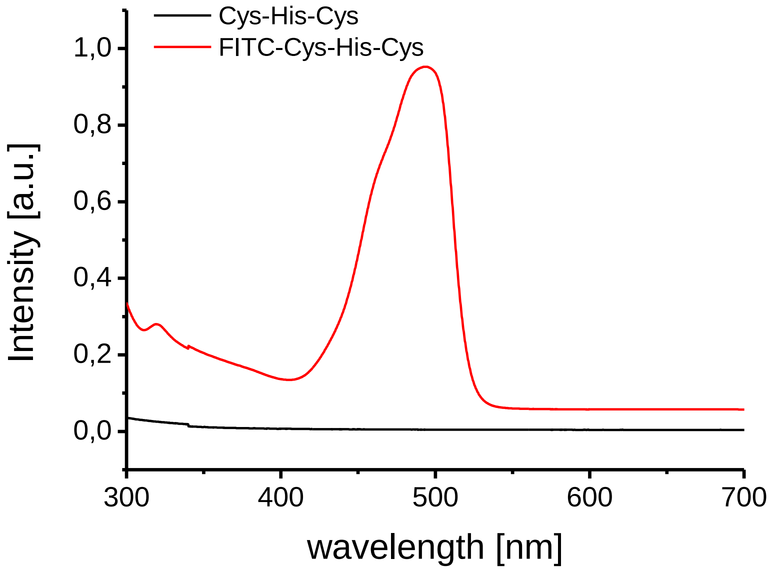

Figure S2: UV–vis spectra of the tripeptides Cys-His-Cys and FITC-Cys-His-Cys.

Water contact angle measurements

Table S2: Water contact angles for epoxide- and carbohydrate-terminated SAMs.

	**Θ stat. [deg.]**	**Θ adv. [deg.]**	**Θ rec. [deg.]**
**Epoxide SAM**	62 ± 2	61 ± 1	33 ± 2
**Glc SAM**	23 ± 2	27 ± 2	<10
**Gal SAM**	25 ± 2	30 ± 2	12 ± 3
**Man SAM**	27 ± 1	30 ± 1	13 ± 3
**NANA SAM**	26 ± 2	33 ± 2	11 ± 3

X-ray photoelectron spectroscopy

X-ray photoelectron spectroscopy (XPS) of Si-wafers functionalized with an epoxide-terminated SAM and printed with NANA ink using a PEG coated flat stamp are shown in Figure S3 and S4. While nearly no N(1s) signal was detected in the epoxy SAM, a clear N(1s) signal at 400 eV can be observed when the NANA ink is printed. Two nitrogen peaks are identified: a major peak which arises from the amide bonds in the carbohydrate ink and a minor one that is attributed to traces of absorbed triethylammonium salt that could not be removed by rinsing or sonification and which originates from the triethylamine added as an additive in the ink solution. The C(1s) peak shows a splitting into the C–C (285 eV), C–O (287 eV), C=C, C=O and residual epoxide C–O signals (289 eV) as expected for the NANA-terminated SAM.

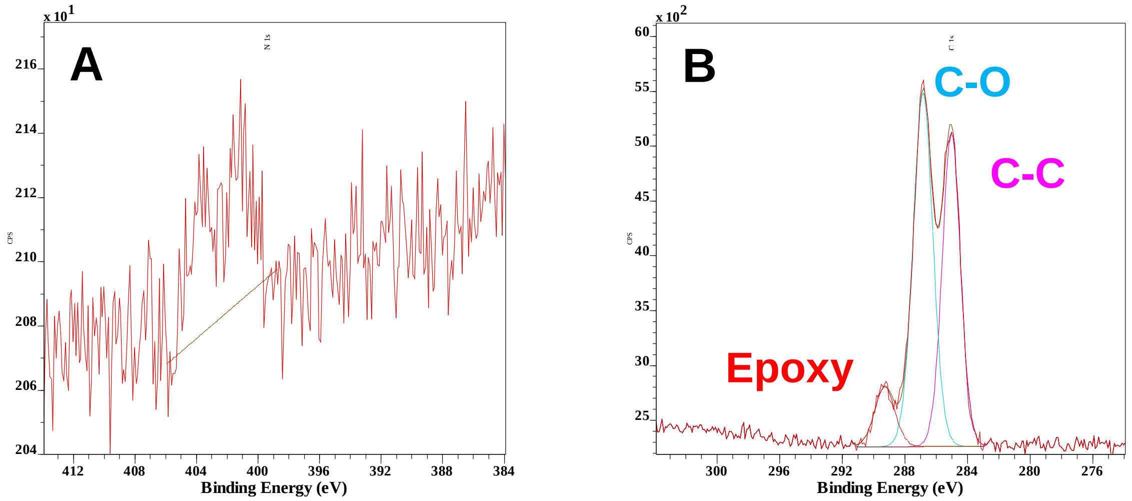

Figure S3: N(1s) (A) and C(1s) (B) region of the XPS spectra of an epoxide-terminated SAM.

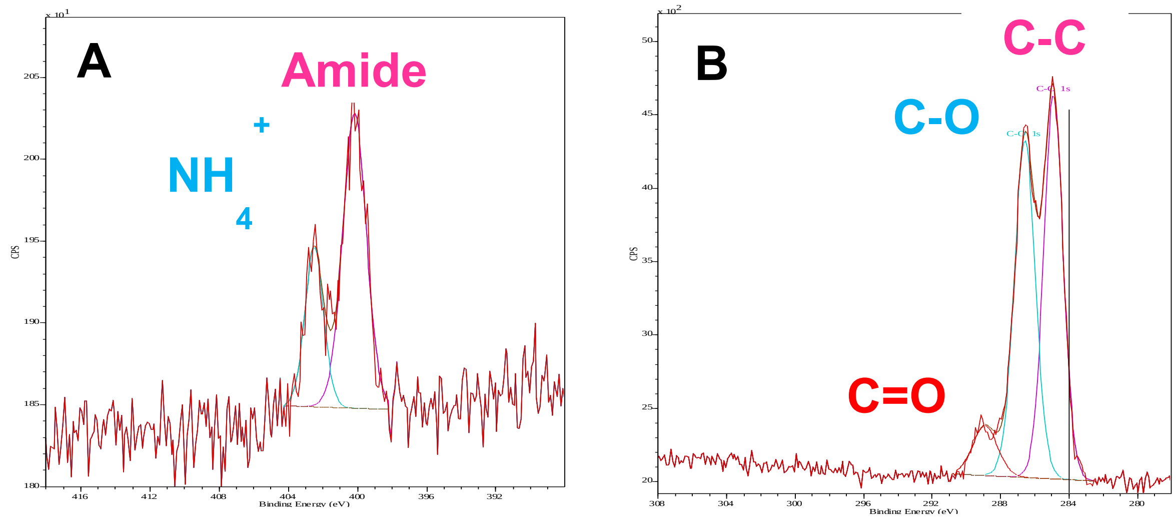

Figure S4: N(1s) (A) and C(1s) (B) region of the XPS spectra of NANA ink printed on an epoxide-terminated SAM.

Surface plasmon resonance (SPR)

In order to confirm the interaction between HisHis and NANA with SPR a commercially available polycarboxylate hydrogel sensor surface was employed. Both the spectrometer and the hydrogel chips were provided by XanTec bioanalytics GmbH, Düsseldorf, Germany. The functionalization of the polycarboxylate hydrogel with amine terminated NANA was performed by *N*-hydroxysuccinimide (NHS) activation and subsequent peptide coupling. All SPR experiments were carried out in NH_4CO_3 buffer (100 mM, pH 7.8) with a flow rate of 10 µL/min at 25 °C. HisHis (0.5–2.0 mM in NH_4CO_3 buffer) was applied to the NANA functionalized surface for 10 min. A small but significant SPR signal increase was observed upon the addition of HisHis. The initial rate and extent of surface binding correlated with the concentration of HisHis (0.5–2.0 mM) applied to the sensor. However, it was not possible to obtain sufficiently reproducible data to perform a quantitative analysis of the peptide–carbohydrate interaction. The poor quality of the SPR signal is certainly due to the low molecular weight of HisHis which limits any further SPR investigations.

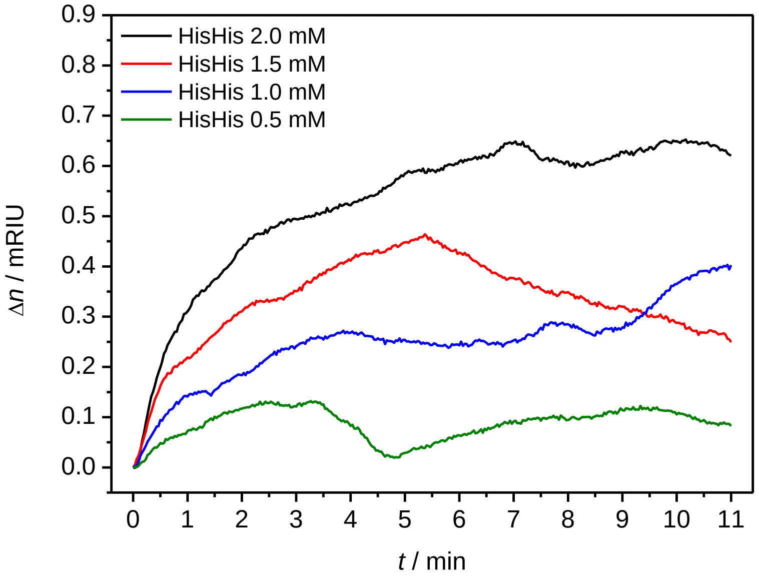

Figure S5: SPR sensogram for HisHis applied to a hydrogel sensor surface functionalized with NANA. In each measurement, the blank reference was substracted from the observed signal trace.
